# Ambient air pollution and cardiovascular diseases: An umbrella review of systematic reviews and meta‐analyses

**DOI:** 10.1111/joim.13467

**Published:** 2022-03-08

**Authors:** Jeroen de Bont, Suganthi Jaganathan, Marcus Dahlquist, Åsa Persson, Massimo Stafoggia, Petter Ljungman

**Affiliations:** ^1^ Institute of Environmental Medicine Karolinska Institutet Stockholm Sweden; ^2^ Centre for Environmental Health Public Health Foundation of India Delhi‐NCR India; ^3^ Centre for Chronic Disease Control New Delhi India; ^4^ Department of Epidemiology Lazio Region Health Service Rome Italy; ^5^ Department of Cardiology Danderyd University Hospital Danderyd Sweden

**Keywords:** ambient air pollution, cardiovascular diseases, nitrogen oxides, particulate matter, umbrella review

## Abstract

The available evidence on the effects of ambient air pollution on cardiovascular diseases (CVDs) has increased substantially. In this umbrella review, we summarized the current epidemiological evidence from systematic reviews and meta‐analyses linking ambient air pollution and CVDs, with a focus on geographical differences and vulnerable subpopulations. We performed a search strategy through multiple databases including articles between 2010 and 31 January 2021. We performed a quality assessment and evaluated the strength of evidence. Of the 56 included reviews, the most studied outcomes were stroke (22 reviews), all‐cause CVD mortality, and morbidity (19). The strongest evidence was found between higher short‐ and long‐term ambient air pollution exposure and all‐cause CVD mortality and morbidity, stroke, blood pressure, and ischemic heart diseases (IHD). Short‐term exposures to particulate matter <2.5 μm (PM_2.5_), <10 μm (PM_10_), and nitrogen oxides (NO*_x_*) were consistently associated with increased risks of hypertension and triggering of myocardial infarction (MI), and stroke (fatal and nonfatal). Long‐term exposures of PM_2.5_ were largely associated with increased risk of atherosclerosis, incident MI, hypertension, and incident stroke and stroke mortality. Few reviews evaluated other CVD outcomes including arrhythmias, atrial fibrillation, or heart failure but they generally reported positive statistical associations. Stronger associations were found in Asian countries and vulnerable subpopulations, especially among the elderly, cardiac patients, and people with higher weight status. Consistent with experimental data, this comprehensive umbrella review found strong evidence that higher levels of ambient air pollution increase the risk of CVDs, especially all‐cause CVD mortality, stroke, and IHD. These results emphasize the importance of reducing the alarming levels of air pollution across the globe, especially in Asia, and among vulnerable subpopulations.

## Graphical abstract

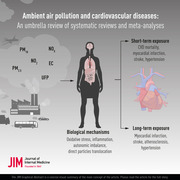

## Introduction

Ambient air pollution is considered the foremost environmental risk factor of mortality and morbidity worldwide [1]. Recent evidence from the Global Burden of Disease (GBD) study estimated that ambient air pollution caused 4.2 million deaths (7.6% of total global mortality) and 103.1 million disability‐adjusted life‐years (DALYs) (4.2% of global DALYs) in 2015 [1]. More specifically, ambient air pollution has been associated with adverse respiratory diseases, decreased lung function, cancer, obesity, diabetes, and cardiovascular diseases (CVD) among adults [2, 3, 4]. In fact, the GBD in 2015 reported that air pollutants accounted for 19% of all cardiovascular deaths [1].

**Fig. 1:**
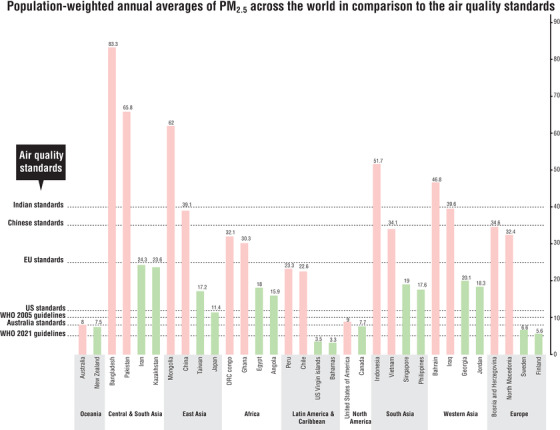
Graph showing population‐weighted annual PM_2.5_ (where PM is particulate matter) averages (2019) and air quality standards (Note: presenting the two most polluted and two least polluted countries in each region) [8].

Air pollution is a complex mixture of particles and gases whose physical and chemical composition, origin, and toxicity differ spatially and temporally [5, 6]. Air pollutants in the solid phase are usually classified according to their size—PM_2.5_ are particulate matter (PM) less than 2.5 μm in diameter, PM_10_ are less than 10 μm, PM_coarse_ are between 2.5 and 10 μm, and ultrafine particles (UFP) are less than 0.1 μm. Smaller sizes of PM have been associated with more adverse effects on human health as they can penetrate more deeply into the respiratory tract [2]. Particles may have substantially different chemical and physical properties and emissions can vary between locations and over time [6]. In urban areas, PM_2.5_ and UFP are directly generated by combustion processes in vehicles, industry and energy production, and from domestic heating, whereas PM_10_ and PM_coarse_ are composed largely of crustal material, sea salt, and biological materials [5, 6] (Fig. 1). Motor vehicles and industrial plants are also important sources of gaseous air pollutants, such as oxides of nitrogen (NO_x_) derived from combustion processes of burning fossil fuels at high temperatures. Nitrogen dioxide (NO_2_) is the secondary pollutant of nitrogen oxides that reacts rapidly with oxygen to form NO_2_. In nonurban or rural areas, NO_2_ is usually at its lowest levels as it is consumed in secondary processes involving gaseous precursors to generate fine particles. Overall, there seems to be no safe threshold for air pollution levels. Even in countries with low levels of air pollution, there is evidence of associations with cardiovascular health [7]. Interestingly, irrespective of the body of evidence available, air pollution guidelines vary substantially across the globe (Fig. 1). The lowest annual thresholds of PM_2.5_ are the Australian air quality guidelines (8 μg/m^3^), whereas China and India have much higher thresholds (35 and 40 μg/m^3^, respectively). In many countries, especially in low‐and‐middle‐income countries (LMICs), the health effects of long‐term exposure to air pollution are yet to be studied in detail and desired thresholds for air quality are routinely violated [8]. On 22 September 2021, the World Health Organization released revised air quality guidelines based on newer evidence and recommended more stringent guidelines with annual thresholds for PM_2.5_ of 5 μg/m^3^ (a reduction from earlier guidelines of 10 μg/m^3^), further highlighting the urgency to improve air quality across the globe.

The available evidence for CVD effects of air pollution has increased substantially during the last decade [9, 10]. Current reviews have been limited to specific aspects of air pollution and CVD. To date, we lack an umbrella review that has synthesized all the available evidence from systematic reviews and meta‐analysis investigating the effect of PM and NOx on multiple cardiovascular outcomes including short and long‐term studies. Additionally, the levels of ambient air pollution have increased rapidly in LMICs during the last decades, but there are very few studies from such high concentration areas. Hence, more studies in LMICs are needed owing to substantial differences between pollutant sources, composition, spatial‐temporal variability, population characteristics, and access to health care compared with high‐income countries. The effects of air pollution are variable for different population subgroups, owing to age, gender, socio‐economic status, and occupation [11].

In this umbrella review, we aimed to summarize the current epidemiological evidence from systematic reviews and meta‐analyses linking ambient air pollution (PM and NO_x_) and multiple CVDs. We additionally tried to review evidence of heterogenous effects that exist based on geography and among vulnerable subpopulations.

## Methods

### Search strategy, inclusion, and exclusion of reviews

We developed the umbrella review in accordance with the reporting guidance in the Preferred Reporting Items for Systematic Reviews and Meta‐Analyses Protocols (PRISMA‐P) (Table S1). The protocol was registered in the Prospero database (ID: CRD42021250209). We performed a comprehensive search in PubMed, EMBASE and Web of Science for articles published between 1 January 2010 and 31 January 2021, limited to articles published in English (Table S2). The search strategy identified systematic reviews and meta‐analyses of observational studies in humans of different study designs (cohort, case‐crossover, cross‐sectional, and time‐series) evaluating the short‐ and long‐term effect of ambient air pollution on multiple CVDs. We focused on the following exposures: PM (PM_10_, PM_2.5_, PM_coarse_, elemental carbon [EC], and UFP) and nitrogen oxides (NO_x_ and NO_2_); and the following CVD outcomes: all‐cause CVD mortality and morbidity, ischemic heart disease (IHD; in this review, we considered coronary heart disease as part of IHD), myocardial infarction (MI), atherosclerosis, arterial stiffness, blood pressure and hypertension, heart failure, stroke and cerebrovascular diseases, arrhythmias, atrial fibrillation, and cardiac arrest. We excluded reviews that did not specify the following: a research question, search strategy, the databases used, inclusion/exclusion criteria, screening methods, quality assessment of included studies’ quality, and information about data synthesis. We excluded reviews related to ozone, sulphur dioxide, carbon monoxide, indoor air pollution, occupational exposures, transboundary haze/desert storms, intervention studies, and experimental studies. We further excluded nonepidemiological reviews/narrative reviews and reviews that focussed on other outcomes, including metabolic disorders, respiratory conditions, congenital heart diseases, venous thrombosis, and coagulation markers. Two researchers (S.J. and J.D.B.) screened independently the titles and abstracts and conducted full‐text screening for potential inclusion. Any disagreement was resolved in consensus with the rest of the team (M.D., Å.P., M.S., and P.L.).

The data were extracted in duplicate (S.J. and J.D.B.) and reverted to the original article in case of disagreements. The following characteristics were extracted: citation details, type of review, participation details, study setting and context, date range of database searching, number of included studies (types of study and country of origin), main exposures, main outcomes, main results, results pertaining to LMICs, and vulnerable populations (Table S3). For meta‐analytical reviews, we additionally extracted the summary meta‐analytic estimate and corresponding 95% confidence interval (CI), and heterogeneity measure. The effect estimates and CI were converted to percentage excess risk of odds ratio (OR)/relative risk (RR)/hazard ratio (HR; % excess risk = [OR/HR/RR − 1] × 100) and were harmonized to a 10 μg/m^3^ increase of air pollution to enable comparison. For systematic reviews, we summarized the number of studies that found statistically significant associations and the direction of the association estimates.

### Quality assessment

To assess the methodological quality of the included systematic reviews and meta‐analyses, we considered the critical domains of the AMSTAR‐2 (A MeaSurement Tool to Assess Systematic Reviews) checklist [12]. The AMSTAR‐2 quality assessment tool has a 16‐item or domain checklist. Seven of these items were considered critical: (1) protocol registered before starting the review, (2) adequacy of the literature search, (3) justification for excluding individual studies, (4) risk of bias from individual studies included in the review, (5) appropriateness of meta‐analytical methods, (6) consideration of risk of bias when interpreting the results of the review, and (7) assessment of presence and likely impact of publication bias. The quality was rated as low (when only 1 critical domain was met), moderate (between 2 and 5 critical domains were met), and high (6 or all critical domains were met). To evaluate the strength of evidence, we calculated a percentage by dividing the number of meta‐analyses reporting positively significant associations among the total numbers of meta‐analyses for each specific CVD outcome and temporality. Then, we classified the strength of evidence in the following categories: (1) sufficient, evidence is sufficiently conclusive (>75% of the meta‐analyses have shown a statistical and positive significant association); (2) limited, positive associations but not conclusive (>50% to ≤75% of the meta‐analyses have shown statistically significant associations); (3) inadequate, positive association reported in less than 50% of meta‐analyses (>25% to ≤50%); (4) insufficient, no association reported in one or more meta‐analyses (0% to ≤25%); and (5) no studies, no studies have been conducted.

## Results

### Descriptive results

**Fig. 2:**
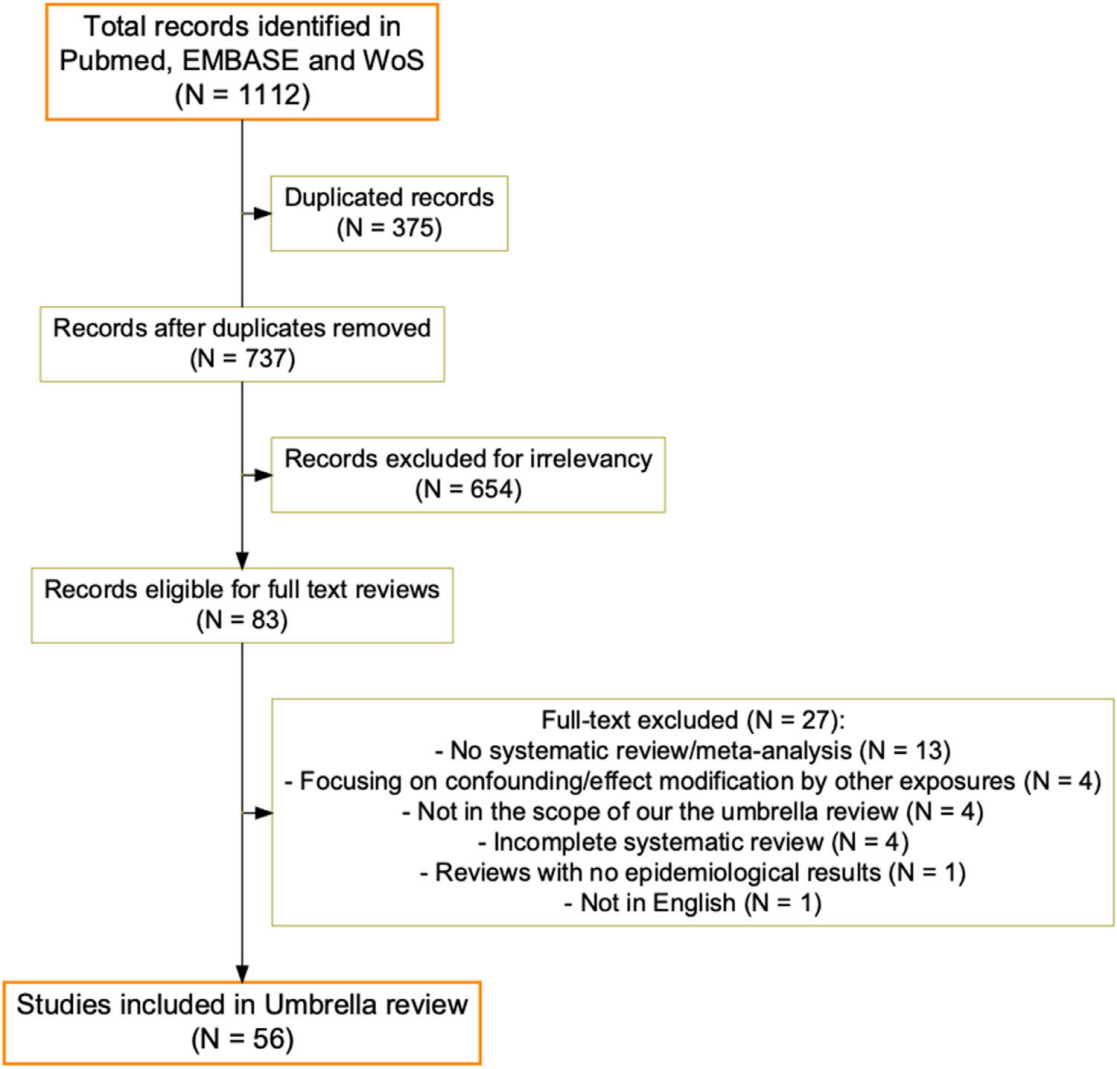
Flowchart of the included reviews.

We identified a total of 1112 publications across the three different databases (Fig. 2). After screening titles and abstracts, we excluded publications for duplicates (*N* = 375) and irrelevant (*N* = 654) publications and identified a total of 83 publications eligible for full‐text reading. We further excluded reviews that were not a systematic review and/or meta‐analysis (13 publications), discussed confounding or effect modification by other exposures rather than the exposure of our interest (4), were not in the scope of our umbrella review (4), were incomplete systematic reviews (4), were reviews lacking epidemiological results (1), or were not in English (1). We finally included a total of 10 systematic reviews and 46 meta‐analyses in this umbrella review (Fig. 2 and Table S3). The most common outcomes were stroke (22 reviews) and all‐cause CVD mortality and morbidity (21) and less common outcomes were cardiac arrest (3) and arterial stiffness (1) (Table 1). A total of 44 reviews focused on short‐term effects and 33 on long‐term effects, and 52 and 26 reviews focused on the effect of PM and nitrogen oxides, respectively (Table 1).

**Table 1:** Summary of included reviews by CVD outcomes, exposure temporality, air pollutants, and percentage of studies in LMICs

				% Studies in LMICs (average)	
CVD outcomes	Exposure duration^a^ Short term (*N* = 44) Long term (*N* = 33)	PM reviews (*N* = 52)^b^ (No. of studies)	NO_x_ reviews (*N* = 46)^b^ (No. of studies)	PM	NO_x_
All‐cause CVD mortality and morbidity	Short term	10 (237)	4 (138)	50.4	65.0
	Long term	10 (178)	6 (73)	12.4	53.3
Ischemic heart disease and myocardial infarction	Short term	9 (171)	4 (121)	10.8	29.1
	Long term	9 (198)	3 (36)	25.0	0.0
Atherosclerosis and arterial stiffness	Short term	1 (13)	1 (2)	0.0	0.0
	Long term	5 (67)	0 (0)	5.1	0.0
Blood pressure and hypertension	Short term	6 (300)	3 (26)	27.5	0.0
	Long term	8 (344)	3 (121)	36.4	0.0
Heart failure	Short term	3 (69)	3 (23)	11.4	0.0
	Long term	1 (9)	1 (3)	0.0	0.0
Stroke	Short term	12 (763)	7 (327)	29.8	0.0
	Long term	11 (301)	4 (51)	15.0	0.0
Arrhythmias, atrial fibrillation, and cardiac arrest	Short term	11 (141)	8 (54)	19.8	0.0
	Long term	2 (15)	2 (5)	0.0	0.0

Abbreviations: CVD, cardiovascular disease; LMICs, low‐and‐middle‐income countries; NO_x_, nitrogen oxides; PM, particulate matter.

^a^ Mutually inclusive/not mutually exclusive/overlapping categories.

^b^ Includes both systematic reviews and meta‐analyses.

### All‐cause CVD mortality and morbidity

**Fig. 3:**
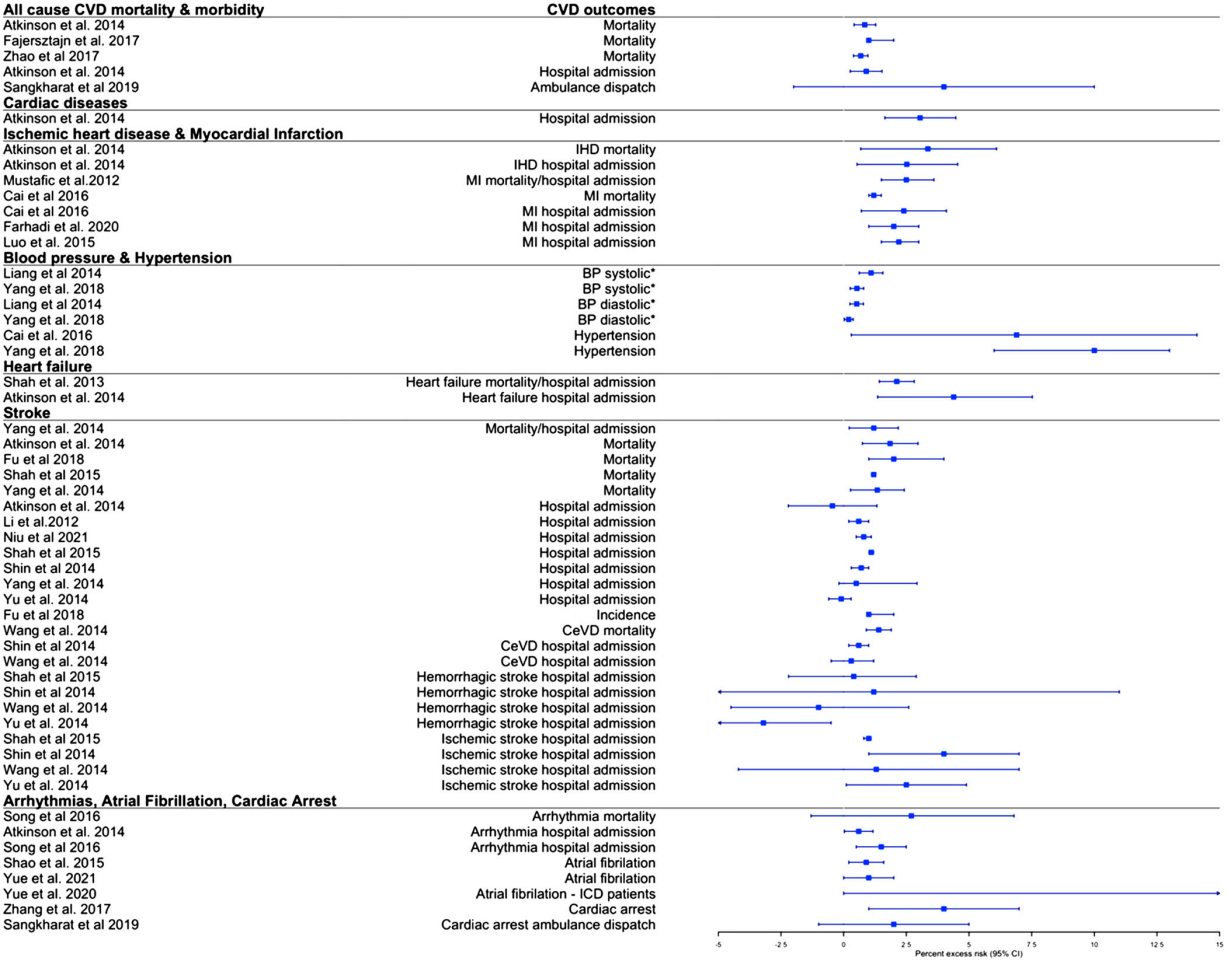
Effect estimates of the association between short‐term exposure to PM_2.5_ and CVDs. Note: we selected the main effect estimate from the meta‐analyses if multiple effect estimates were available for each CVD outcome in the same meta‐analysis. Effect estimates are estimated per 10 μg/m^3^ range increase in PM_2.5._ Abbreviations: BP, blood pressure; CeVD, cerebrovascular diseases; CVD, cardiovascular diseases; ICD, implantable cardioverter defibrillator; IHD, ischemic heart diseases; MI, myocardial infarction; PM, particulate matter. ^*^Beta coefficient (linear regression) for change in systolic and diastolic values per increase of PM_2.5_.

**Fig. 4:**
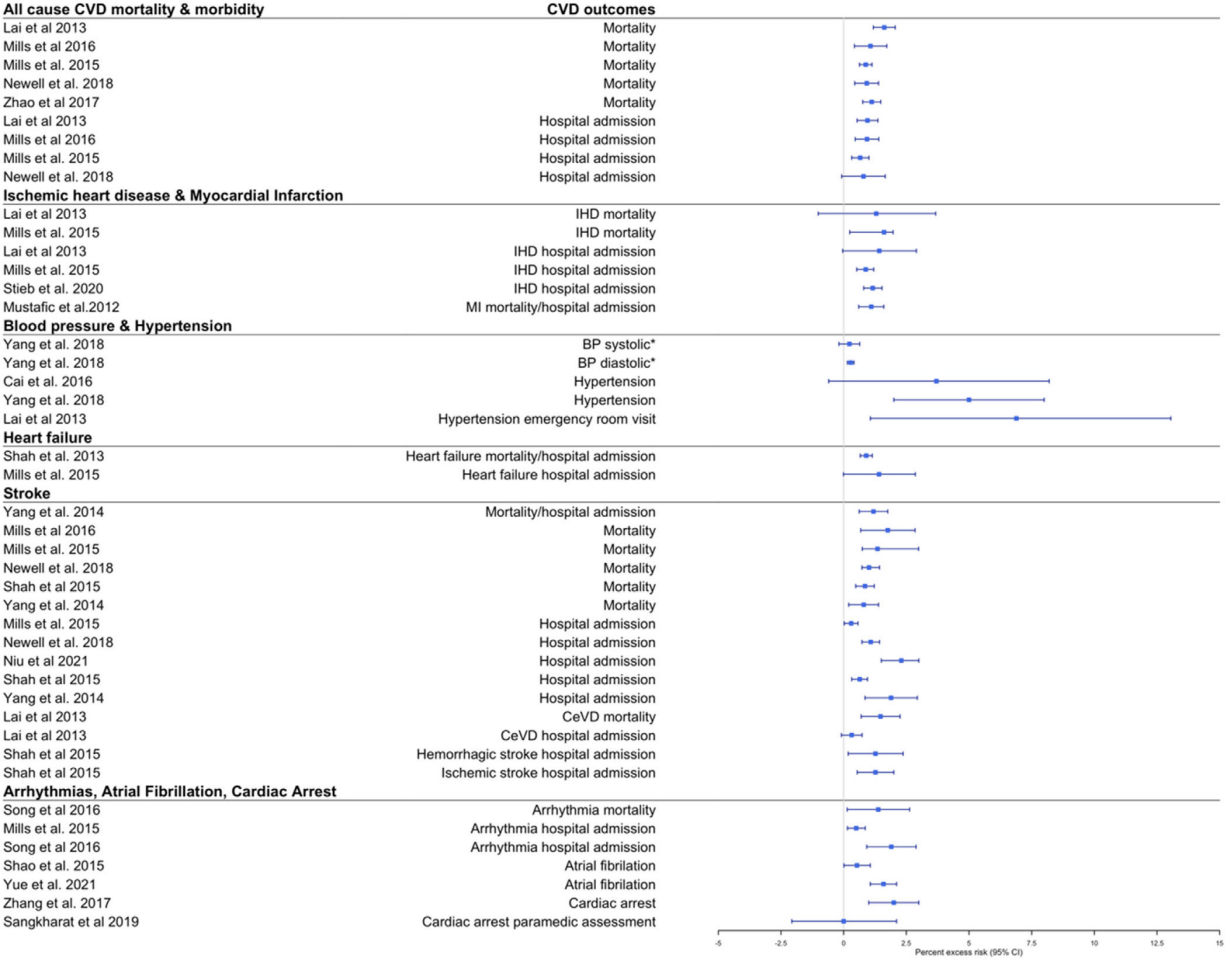
Effect estimates of the association between short‐term exposure to NO_x_/NO_2_ and CVDs. Note: we selected the main effect estimate from the meta‐analyses if multiple effect estimates were available for each CVD outcome in the same meta‐analysis. Effect estimates are estimated per 10 μg/m^3^ range increase in NO_x_/NO_2._ Abbreviations: BP, blood pressure; CeVD, cerebrovascular diseases; CVD, cardiovascular diseases; IHD, ischemic heart diseases; MI, myocardial infarction. ^*^Beta coefficient (linear regression) for change in systolic and diastolic values per increase of NO_x_/NO_2_.

In total, four systematic reviews and 17 meta‐analyses were published between 2010 and 2020 and evaluated the short‐ and long‐term exposures of ambient air pollution on all‐cause CVD mortality and morbidity [13, 14, 15, 16, 17, 18, 19, 20, 21, 22, 23, 24, 25, 26, 27, 28, 29, 30, 31, 32, 33]. From the meta‐analyses, increased short‐term exposure to PM_2.5_ (3 meta‐analytic estimates out of 3), PM_10_ (2/2), and NO_x_ (5/5) were all statistically significantly associated with all‐cause CVD mortality (Figs 3, 4, and S1). Percent excess risk per 10 μg/m^3^ increase ranged from 0.64 (0.39; 0.97) to 1.00 (1.00; 2.00) for PM_2.5_, from 0.39 (0.26; 0.53) to 0.49 (0.35, 0.63) for PM_10_, and from 0.88 (0.63; 1.13) to 1.62 (1.18; 2.06) for NO_x_. From the meta‐analyses reporting all‐cause CVD morbidity (hospital admissions), there were statistically significant positive associations for short‐term exposure to PM_2.5_ (1/2), PM_10_ (2/2), and NO_x_ (3/4) (Figs 3, 4, and S1). In the systematic reviews, all effect estimates from the individual studies were associated with an increased CVD mortality (16 out of 18 individual studies) and morbidity (8/11) for PM_2.5_, but some were not statistically significant (Table S6).

**Fig. 5:**
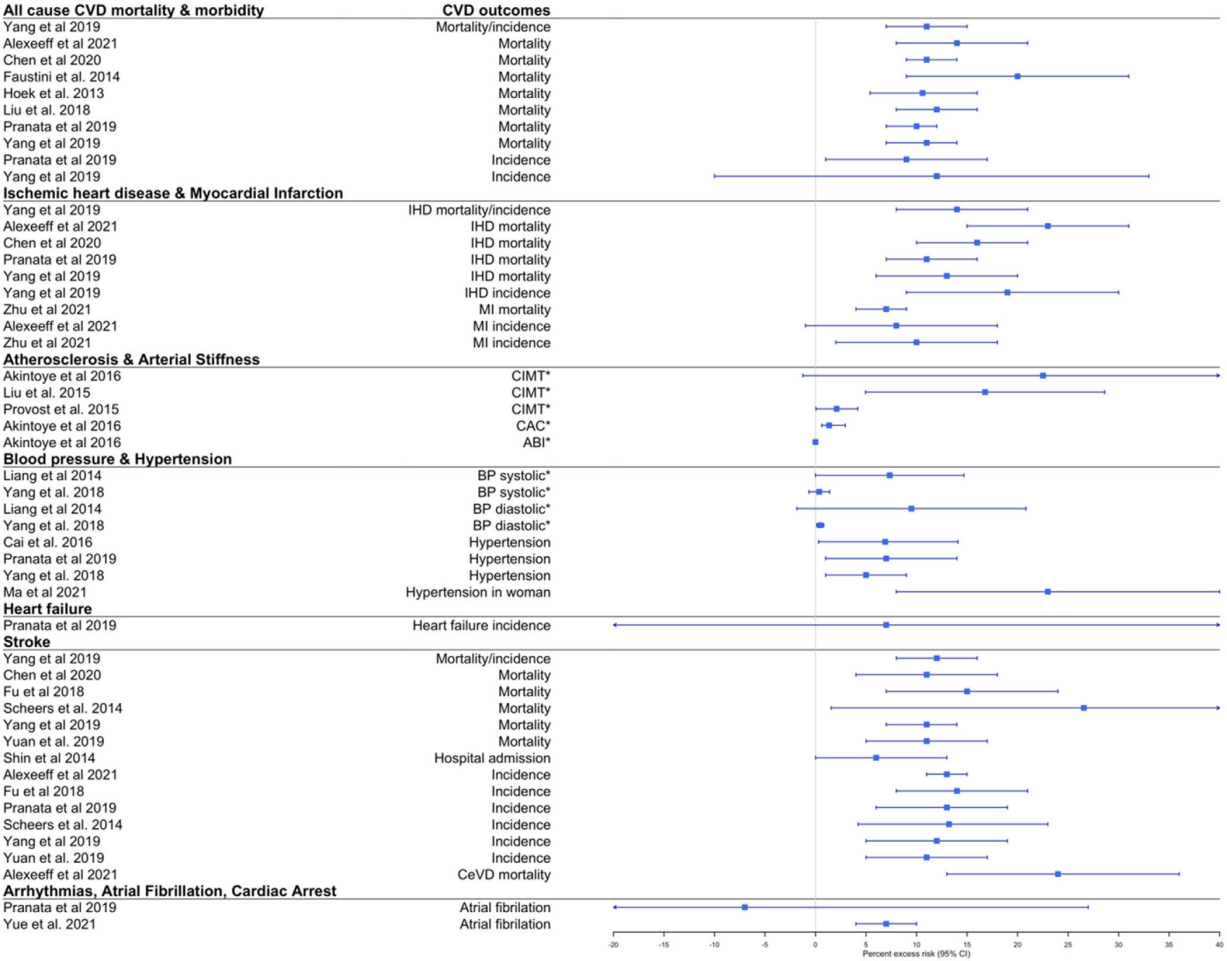
Effect estimates of the association between long‐term exposure to PM_2.5_ and CVDs. Note: we selected the main effect estimate from the meta‐analyses if multiple effect estimates were available for each CVD outcome in the same meta‐analysis. Effect estimates are estimated per 10 μg/m^3^ range increase in PM_2.5._ Abbreviations: ABI, ankle‐brachial index, BP, blood pressure; CAC, coronary artery calcification; CeVD, cerebrovascular diseases; CIMT, carotid intima‐media thickness test; CVD, cardiovascular diseases; IHD, ischemic heart diseases; MI, myocardial infarction; PM, particulate matter. ^*^Beta coefficient (linear regression) for change in systolic, diastolic, CIMT, CAC, or ABI values per increase of PM_2.5_.

**Fig. 6:**
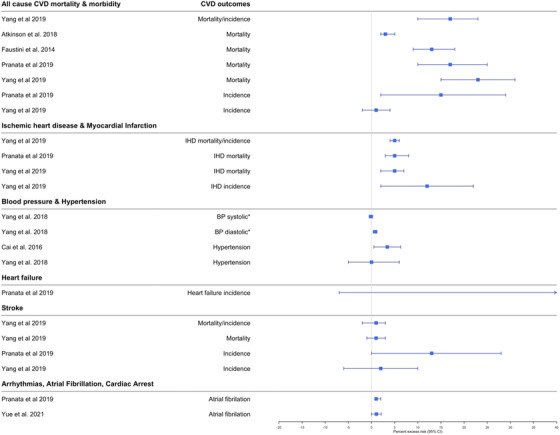
Effect estimates of the association between long‐term exposure to NO_x_/NO_2_ and CVDs. Note: we selected the main effect estimate from the meta‐analyses if multiple effect estimates were available for each CVD outcome in the same meta‐analysis. Effect estimates are estimated per 10 μg/m^3^ range increase in NO_x_/NO_2_. Abbreviations: BP, blood pressure; CVD, cardiovascular diseases; IHD, ischemic heart diseases. ^*^Beta coefficient (linear regression) for change in systolic or diastolic values per increase of NO_x_/NO_2_.

Long‐term exposure to PM_2.5_ (7/7) and NO_x_ (4/4) was consistently associated with all‐cause CVD mortality, but to a lesser extent for PM_10_ (2/4) (Figs 5, 6, and S5). Percent excess risk per 10 μg/m^3^ increase ranged from 11.00 (7.00; 14.00) to 20.00 (9.00; 31.00) for PM_2.5_ and from 3.00 (2.00; 5.00) to 23.00 (15.00; 31.00) for NO_x_ (Figs 5, 6, and S2). Two meta‐analysis reviews evaluated the association between the air pollutants and all‐cause CVD incidence with meta‐analytical effect estimates indicating higher risks, but mostly not statistically significant. A similar consistent pattern with significant positive associations was also observed in the systematic reviews although based on a few number of studies (Table S7).

### Ischemic heart disease and myocardial infarction

A total of 10 and seven meta‐analyses evaluated the short‐ and long‐term exposure to PM and NO_x_ on IHD and MI, respectively [13, 16, 20, 22, 24, 25, 28, 29, 30, 33, 34, 35, 36, 37, 38, 39, 40]. For both IHD and MI, two systematic reviews were included.

The short‐term exposure to PM_2.5_ (3/3) and NO_x_ (3/5) was associated with IHD mortality and hospital admission. Percent excess risk per 10 μg/m^3^ increase ranged from 2.50 (1.50; 3.60) to 3.36 (0.68; 6.10) for PM_2.5_ and from 0.88 (0.52; 1.20) to 1.42 (−0.04; 2.90) for NO_x_ (Figs 3 and 4). For PM_10_, one out of two meta‐analyses found a statistically significant association with increased IHD mortality/hospital admission. In the systematic reviews, most of the individual reviews concluded that air pollutants were associated with increased IHD mortality (Table S6), especially in the Zhang and Routledge [25] review that focused only on studies conducted in China (Table S3). Regarding the long‐term exposures, nine out of 10 meta‐analytic estimates found that increased levels of PM_2.5_ (4/4), PM_10_ (2/3), and NO_x_ (2/2) were associated with increased risk of IHD mortality (Figs 5, 6, and S2). Higher long‐term exposures to PM_2.5_ (3/3), PM_10_ (1/3), and NO_x_ (2/3) were also associated with incident IHD.

Focussing on reviews of air pollution and MI, short‐term exposures to PM_2.5_ (4/4), PM_10_ (4/4), and NO_x_ (1/1) were associated with increased triggering of MI mortality and hospital admissions (Figs 3, 4, and S1). Percent excess risk per 10 μg/m^3^ increase ranged from 1.20 (1.00; 1.50) to 2.40 (0.70; 4.10) for PM_2.5_, from 0.50 (0.10; 0.80) to 1.10 (0.60; 1.60) for PM_10_, and 1.10 (0.60; 1.60) for NO_x_. Two out of three meta‐analyses reported a statistically significant association with increased long‐term exposure to PM_2.5_ and increased risk of MI mortality/incidence (Figs 5, 6, and S2). No meta‐analyses evaluated the effect of long‐term effect of PM_10_ and NO_x_ on MI. From the systematic reviews, no conclusive results could be drawn as only a few individual studies were included (between 1 and 3 studies, Table S7).

### Atherosclerosis and arterial stiffness

No systematic reviews or meta‐analyses evaluated the short‐term effects of air pollution on atherosclerosis. One systematic review and three meta‐analyses examined the association between long‐term exposure to PM (PM_2.5_ and PM_10_) and atherosclerosis, but no reviews evaluated the long‐term effect of NO_x_ [41, 42, 43, 44]. The most common measurements to assess atherosclerosis were carotid intima‐media thickness (CIMT), followed by coronary artery calcification (CAC), arterial calcification, and ankle‐brachial index (ABI). Two out of three meta‐analytical estimates demonstrated statistically significant associations between PM_2.5_ and CIMT ranging from 2.09 mm (0.04; 4.48) to 16.79 mm (4.95; 28.63) increased CIMT per year for every 10 μg/m^3^ increase in PM_2.5_ (Fig. 5). No associations were found between PM_10_ and CIMT. In addition, in one meta‐analysis, PM_2.5_ was associated with CAC but not with ABI. In one systematic review [44], the individual observational studies demonstrated that increased levels of PM were statistically associated with CIMT (6 out of 9 individual studies), CIMT progression (4/4), CAC prevalence (4/7), and CAC progression (1/2) (Table S7).

One systematic review evaluated both short‐ and long‐term exposure to PM_2.5_, PM_10_, and NO_x_ on arterial stiffness (Tables S6 and S7) [45]. They reported that increased levels of PM (PM_2.5_ and PM_10_) and NO_x_ were associated with increased arterial stiffness and pulse wave reflection, but no statistically significant association was observed for brachial‐ankle pulse wave velocity.

### Blood pressure and hypertension

A total of 10 reviews (6 meta‐analyses) examined the short and long‐term effect of PM (PM_2.5_, PM_10_, black carbon/EC, UFP, and PM_coarse_) on blood pressure and hypertension, and only three studies additionally explored the association with NO_x_ [19, 20, 24, 27, 46, 47, 48, 49, 50, 51]. In the short‐term exposure studies, higher exposures to PM_2.5_ (2 meta‐analytic estimates out of 2), PM_10_ (1/1), and NO_x_ (1/1) were associated with higher diastolic blood pressure, whereas only PM_2.5_ (2/2) was also associated with higher systolic blood pressure (Figs 3, 4, and S1). The increase of mmHg in diastolic blood pressure per 10 μg/m^3^ increase of PM_2.5_ ranged from 0.15 mmHg (0.01; 0.19) to 0.52 mmHg (0.25; 0.79). Further, increased short‐term exposure to PM_2.5_ (2/2), PM_10_ (3/3), and NO_x_ (2/3) was associated with an increased risk of hypertension. In the systematic reviews, short‐term exposure to EC was not consistently associated with blood pressure, whereas another review found a consistently small positive association between short‐term exposure to UFP and systolic and diastolic blood pressure (Table S6).

Similar to the short‐term effects, a meta‐analysis of long‐term exposures demonstrated significant associations between higher PM_2.5_ (1/2), PM_10_ (1/1), and NO_2_ (1/1) and increased diastolic blood pressure but not systolic blood pressure (Figs 5, 6, and S2). In addition, long‐term exposure to PM_2.5_ was associated with incident hypertension in all included meta‐analyses (3/3). The percentage risk of incident hypertension ranged from 5.00 (1.00; 9.00) to 7.00 (1.00; 14.00) per 10 μg/m^3^ increase in PM_2.5_ (Fig. 5). PM_10_ (1/2) and NO_x_ (1/2) were less consistently associated with incident hypertension. The meta‐analytic excess risk was even larger in a study that focused on the long‐term risk of PM_2.5_ on hypertension in women (per cent excess risk per 10 μg/m^3^ increase in PM_2.5_ = 23.00 [8.00; 40.00]). In the systematic reviews, one review reported that 14 out of 19 included studies found a positive association between EC and systolic and diastolic blood pressure, and another found in two studies a positive association between PM_10_ and hypertension in LMICs (Table S7).

### Heart failure

Five meta‐analyses studied the association between short‐ and long‐term exposure to air pollution on heart failure [13, 20, 22, 24, 52]. The short‐term exposures to PM_2.5_ (2 meta‐analytic estimates out of 2), PM_10_ (1/1), and NO_x_ (2/2) were associated with heart failure mortality and hospital admissions (Figs 3, 4, and S1). Percent excess risk per 10 μg/m^3^ ranged from 2.12 (1.42; 2.82) to 4.39 (1.35; 7.53) for PM_2.5_, 2.12 (1.42; 2.82) for PM_10_, and from 0.90 (0.66; 1.14) to 2.41 (−0.01; 2.86) for NO_x_. In contrast, the one meta‐analysis [24] that evaluated the long‐term effect of PM_2.5_, PM_10_, and NO_x_ on heart failure demonstrated uninformative associations with very large CIs (Figs 5, 6, and S2).

### Stroke

In total, two systematic reviews and 20 meta‐analyses evaluated the short‐ and long‐term effects of ambient air pollution on stroke mortality and morbidity, resulting in the most studied CVD outcome during our research period [13, 16, 20, 22, 23, 24, 25, 28, 29, 30, 31, 33, 53, 54, 55, 56, 57, 58, 59, 60, 61, 62]. Increased short‐term exposure to PM_2.5_ (5 meta‐analytic estimates out of 5), PM_10_ (2/2), and NO_x_ (5/6) was statistically associated with increased stroke mortality (Figs 3, 4, and S1). Regarding stroke hospital admission, statistically significant associations were found for PM_2.5_ (4/7), PM_10_ (4/4), and NO_x_ (5/5). Similar results were found in a systematic review (Table S6). All pollutants were associated with increased cerebrovascular mortality and, to a large extent, associated with cerebrovascular hospital admission. Regarding specific types of strokes, one meta‐analysis found that NO_x_ was associated with hospital admissions for hemorrhagic and ischemic stroke, but no clear associations were found between short‐term exposures to PM_2.5_ and PM_10_ and specific stroke types (Figs 3, 4, and S1).

Long‐term exposure to PM_2.5_ was consistently associated with increased stroke mortality (5/5) and stroke incidence (7/7) in meta‐analyses (Fig. 6) and one systematic review (Table S7). The percent excess risk for long‐term exposure to PM_2.5_ per 10 μg/m^3^ increase ranged from 11.00 (4.00; 18.00) to 26.56 (1.54; 57.75) for increased stroke mortality and from 11.00 (5.00; 17.00) to 14.00 (8.00; 21.00) for increased stroke incidence. No associations were observed between the long‐term effects of PM_10_ and NO_x_, and stroke mortality and incidence (Fig. 6/S2).

### Arrhythmias, atrial fibrillation, and cardiac arrest

Two systematic reviews and four meta‐analyses evaluated the short‐term effect of PM_2.5_, PM_10_, and NO_x_ on arrhythmias (all subtypes grouped together) [13, 20, 22, 34, 47, 63]. No reviews on the long‐term effects of air pollution and arrhythmias were found. PM_2.5_ (2 meta‐analytic estimates out of 2), PM_10_ (1/1), and NO_2_ (1/1) were associated with increased hospital admission for arrhythmia, and only NO_x_ was additionally associated with increased mortality due to arrhythmias (Figs 3, 4, and S1). Among the systematic reviews, the included studies showed consistent trends of positive estimates between PM and arrhythmias but did not reach statistical significance (Table S6). No reviews on the long‐term effects of air pollution and arrhythmias were found.

There were four meta‐analyses that evaluated the short‐ and long‐term effect of air pollution on atrial fibrillation [24, 64, 65, 66]. Increased short‐term exposure to PM_2.5_ (2/2), PM_10_ (1/1), and NO_2_ (2/2) was associated with increased atrial fibrillation (Figs 3, 4, and S1). Another meta‐analysis [65] found that a 10 μg/m^3^ increase in PM_2.5_ was associated with a 24.00% (0.00; 53.00) excess risk of atrial fibrillation in patients with an implantable cardioverter‐defibrillator (Fig. 3). In addition, increased long‐term exposure to PM_2.5_ (1/2), PM_10_ (1/2), and NO_2_ (2/2) was inconsistently associated with increased atrial fibrillation (Figs 5, 6, and S2).

One systematic review and two meta‐analyses studied the association between short‐term exposure to air pollutants and out‐of‐hospital cardiac arrest (OHCA) [26, 67, 68]. In one meta‐analysis, short‐term exposure to PM_2.5_, PM_10_, and NO_2_ was associated with increased OHCA. The other meta‐analysis focussed only on ambulance dispatch data and found similar results in direction and magnitude, but these were not statistically significant (Figs 3, 4, and S1). Finally, in the systematic review, consistent results were found between short‐term exposure to PM_2.5_ and PM_10_ on OHCA, but not for NO_x_ (Table S6).

### Strength of evidence

Overall, using our a priori definition, we found sufficient strength of evidence for both short‐ and long‐term exposure to PM and increased risk of CVD mortality, IHD, and MI, and for short‐term PM exposure and increased risk of stroke, blood pressure, heart failure, and arrhythmias (Table 2). There was limited evidence supporting an associated risk between long‐term exposure to PM and atherosclerosis or arterial stiffness outcomes and for incident stroke. There was inadequate evidence to support associations between long‐term PM exposure and blood pressure, hypertension, and arrhythmias. Similar to PM, there was sufficient evidence in support of associations between NO_x_ and CVD mortality or morbidity for both short‐ and long‐term exposure. Sufficient evidence also supported associations between short‐term exposure to NO_x_ and stroke and arrhythmias, and long‐term exposure to NO_x_ and IHD and MI. The strength of evidence for NO_x_ is limited or inadequate for blood pressure or hypertension for bothering short and long term. The strength of evidence, however, should be interpreted with caution, as some of the outcome categories (heart failure, atherosclerosis, and arrhythmias) were included in very few reviews/meta‐analyses.

**Table 2:** Strength of evidence of included reviews and meta‐analysis

CVD outcomes	Exposure temporality	Strength of evidence^a^	
		PM (%)	NOx (%)
All‐cause CVD mortality and morbidity	Short term	Sufficient (100)	Sufficient (100)
	Long term	Sufficient (80)	Sufficient (100)
Ischemic heart disease and myocardial infarction	Short term	Sufficient (90)	Limited (60)
	Long term	Sufficient (80)	Sufficient (100)
Atherosclerosis and arterial stiffness	Short term	No reviews	No reviews
	Long term	Limited (60)	No reviews
Blood pressure and hypertension	Short term	Sufficient (90)	Limited (60)
	Long term	Inadequate (50)	Inadequate (50)
Heart failure	Short term	Sufficient (100)	Sufficient (100)
	Long term	No reviews	No reviews
Stroke	Short term	Sufficient (80)	Sufficient (90)
	Long term	Limited (60)	No reviews
Arrhythmias, atrial fibrillation, and cardiac arrest	Short term	Sufficient (100)	Sufficient (100)
	Long term	Inadequate (50)	Inadequate (50)

Abbreviations: CVD, cardiovascular disease; NO_x_, nitrogen oxides; PM, particulate matter.

^a^ Strength of evidence was stratified as follows: sufficient—evidence is sufficiently conclusive (>75% meta‐analyses‐significant association); limited–positive associations but not conclusive (>50% to ≤75% meta‐analyses‐significant associations); inadequate—positive association reported in less than 50% of meta‐analyses (>25% to ≤50%); insufficient—no association reported in one or more meta‐analyses (0% to ≤25%); no reviews—no reviews have been conducted.

### Geographic differences and vulnerable populations

A total of 26 out of the 56 reviews mentioned the presence of different associations of ambient air pollution and CVD outcomes according to geographical locations and vulnerable populations (Table 1).

Among the included reviews and meta‐analyses, a majority of the evidence came from high‐income countries. Very few outcomes had a considerable number of primary studies conducted in LMICs, as described in Table 1. The risk of air pollution exposure for CVD outcomes was likely to be stronger in Asian countries, with higher levels of air pollution compared to North America and Europe (7 out of 9 mentioned higher meta‐analytic estimates), although heterogeneity was higher. In addition, two other meta‐analyses also found stronger associations in more heavily polluted areas around the world (2/2).

Elderly people (4/4), cardiac patients (2/2), and subgroups with higher body mass index status (2/2) were more susceptible to the adverse effects of air pollution. Inconsistent differences were observed across sex (4/7 found higher estimates among women), smoking status (only 1/4 find stronger associations among smokers), and more socio‐economically deprived people (1 out of 2). There were also other reviews suggesting that women in their menopause (1 review), non‐White population (1), and people with diabetes (1) are vulnerable subgroups.

## Discussion

This umbrella review summarized the epidemiological evidence of short‐ and long‐term exposure to PM and NO_x_ and risk of multiple CVDs and indicated, overall, that exposure to increased levels of ambient air pollution was consistently associated with several important adverse cardiovascular health outcomes. We noted the largest amount of evidence for ambient air pollution and all‐cause CVD mortality, all‐cause CVD hospital admission, IHD, blood pressure, and stroke. Short‐term exposure to PM and NO_x_ was consistently associated with MI, hypertension, and stroke mortality and stroke morbidity. By and large, long‐term exposure to PM_2.5_ was associated with increased incidence of MI, atherosclerosis, incident hypertension, incident stroke, and stroke mortality. Few reviews focussed on heart failure, arrhythmias, and atrial fibrillation but most of them reported statistically significant positive associations. The reviews further suggested a stronger impact of air pollution on CVD outcomes in LMICs, especially in Asia where higher levels of air pollution are observed, and among specific population subgroups like the elderly, patients with preexisting CVD, and people with higher body mass index status.

### Mechanistic support for CVD effects of air pollution exposure from animal and experimental studies

**Fig. 7:**
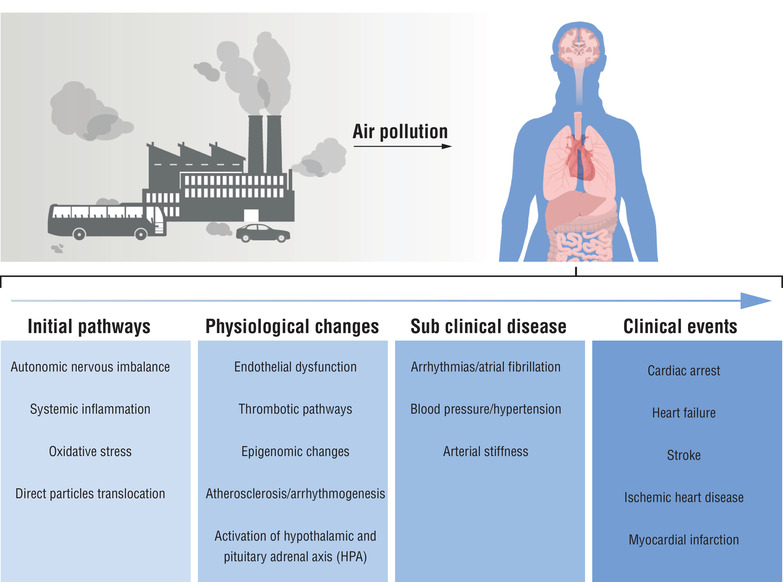
Potential biological pathways between air pollution and cardiovascular diseases.

Studies evaluating the mechanisms underlying the effects of air pollution on CVD have increased substantially during the last decade. The most common initial pathways are (1) oxidative stress and inflammation, (2) autonomic nervous imbalance, and (3) direct particle translocation. These pathways may activate secondary pathways such as endothelial dysfunction, thrombotic pathways, activation of a hypothalamic and pituitary‐adrenal axis (HPA), and epigenomic changes (Fig. 7). The pathways are distinct and may occur at different time points and parts of the body, but they are highly interconnected with effects that may converge at some point to increase the risk of CVD outcomes [69]. In addition, many pathways share similar CVD intermediate endpoints, leading in varying degrees to arrhythmogenesis, increased blood pressure, atherosclerosis development, or increased arterial stiffness. These may in turn increase the risk of developing distinct CVD clinical events, including cardiac arrest, IHD, heart failure, stroke, and finally, CVD mortality. The relevant order or duration of air pollution exposure may activate different pathways at various stages. Some pathways may be activated after short‐term exposures (e.g., autonomic imbalance), and others may be promoted by long‐term exposure (e.g., atherosclerosis). Overall, the influence on the pathways will depend on the type of pollutant, dose and duration of exposure, specific cardiovascular endpoints, and individual health status [70].

#### Ischemic heart disease and myocardial infarction

Controlled human exposures studies and toxicological studies have demonstrated that increased levels of air pollution may increase the sensitivity of the heart to ischemic stress through different pathways [9, 10, 71]. During the last decades, experimental studies have shown that short‐ and long‐term inhalation of PM_2.5_ provokes oxidative stress and systemic inflammation [9, 10]. Oxidative stress is a state where higher levels of free radicals, reactive oxygen species (ROS), and reactive nitrogen species are accumulated in different parts of the body, including the lungs, vascular bed, and even at a local cellular/tissue level. The disruption of redox signaling and excess ROS is suggested to increase adverse biological effects (e.g., lipid/protein/deoxyribonucleic acid [DNA] oxidation and initiation of proinflammatory cascades) and may alter cardiac and vascular function through the disruption of important redox‐sensitive signaling pathways, the depletion of vasodilators and antioxidants, the perturbation of cellular mechanisms, and the oxidation of proteins and lipids [10, 69]. For example, PM_2.5_ was shown to reduce antioxidant capacity in mice and decrease the contractility of cardiomyocytes [72]. Inflammation and oxidative stress are closely interconnected and both lead to a cascade of molecular processes that further affect vascular function and hemostasis [10, 37]. These experimental studies have demonstrated that an increase of PM was associated with increases in both cellular and biochemical markers of pulmonary and systemic inflammation, including interleukin‐6 and C‐reactive protein, among others. The release of cytokines can alter hemostasis and increase the risk of thrombosis [10, 73]. Nitrogen oxides have demonstrated a similar response to that of PM in both animal models and humans, starting with pulmonary inflammation and migration of inflammatory markers causing systemic inflammation and oxidative stress, which can result in activation of cells lining the blood vessels [9]. However, the evidence supporting the biological plausibility of NO_x_ is still considered to be limited and inconsistent [9]. Overall, oxidative stress and systemic inflammation potentially induce several pathological responses (e.g., endothelial dysfunction, vasoconstriction, coagulation) that may increase the risk of IHD and MI, and other CVD outcomes [9, 10, 24, 56]. Indeed, in controlled human exposure and toxicological studies, short‐term exposure to air pollutants has been demonstrated to increase blood viscosity and initiate thrombotic responses and hypercoagulability [9, 10, 37, 74]. PM also causes impaired flow‐mediated dilation, vasoconstriction, temporary coronary occlusion, and ischemia in animal and human experimental studies, all suggested as potential mechanisms leading to cardiovascular morbidity including IHD and MI [8]. Further corroborating this, experimental human and animal model studies suggest progression and changes in atherosclerotic plaques and atherothrombotic events such as MI and ischemic stroke on exposure to air pollution [75].

#### Atherosclerosis and arterial stiffness

The pathways between long‐term exposure to air pollution and, specifically, atherosclerosis can be explained by an interplay between plasma lipoproteins, inflammation, endothelial activation, neutrophil attraction to the endothelium, extravasation, and lipid uptake [10]. Animal studies in mice lacking apolipoprotein necessary for the clearance of fats and cholesterol found that air pollution accelerates atherosclerosis [10, 76]. Another animal study provided evidence for PM_2.5_‐mediated effects on atherosclerotic plaque progression in a genetically susceptible mouse model [77]. In addition, animal and human studies have observed that short‐ and long‐term exposure to air pollution can provoke persistent effects on vascular function, increasing arterial stiffness, impaired conduit artery flow‐mediated dilatation, arteriolar dysfunction, and retinal artery changes [78]. In human experiments, controlled short‐term exposures to PM_2.5_ were associated with decreased brachial artery flow, increased vasoconstrictor endothelin‐1 in blood, and increased levels of vascular endothelial growth factor as a marker for vascular damage [42, 79–81]. Long‐term exposure to air pollution may provoke the depletion of endothelial progenitor cells, explaining vascular remodeling effects [82, 83]. Consistent with these vascular effects, a high concentration of PM has further been associated with increased plaque formation and decreased plaque stability in animal and human studies. Overall, short‐term changes in the endothelial function may provoke changes in vascular tone and stiffness, whereas long‐term changes may lead to increased cardiac afterload, diastolic dysfunction, alteration in coronary flow reserve, and left ventricular hypertrophy and fibrosis [9, 10].

#### Blood pressure and hypertension

As has been previously mentioned, air pollution has been shown to induce inflammation, oxidative stress, and vascular effects such as endothelial dysfunction and arterial stiffness, all known contributors to hypertension. Additionally, daily changes in air pollution may trigger short‐term effects on blood pressure and hypertension by altering the autonomic nervous system (ANS) [10, 48]. Inhalation of particles may stimulate the sympathetic nervous system, increasing the circulating levels of vasoconstrictors, elevating blood pressure, and decreasing the flow to the heart [9, 10]. A study conducted by Wold et al. [72] found that, relative to controls, mice exposed long term to PM_2.5_ had a statistically significant increase in systolic and diastolic blood pressure and mean arterial pressure, while pulse pressure decreased relative to controls. Although less studied, some toxicological evidence suggests that NO_2_ may increase arterial stiffness, increase markers for CVD stability and progression and dyslipidemia, decrease heart‐rate variability (HRV), and reduce heart rate, but whether this reflects an independent effect of NO_2_ is still not clearly distinguished in the available body of epidemiologic and toxicological evidence [9].

#### Heart failure

Emerging evidence suggests that prenatal and postnatal PM_2.5_ exposure can result in heart failure later in life [84]. A study conducted by Gorr et al. in 2014 exposed female mice to PM_2.5_ during pregnancy and found an association with reduced left ventricular fractional shortening with greater ventricular systolic diameter, reduced ejection fraction, and other indicators of cardiac dysfunction when compared to control mice during a postnatal time [85]. A similar study confirmed these findings of ventricular dysfunction as well as prolonged increased action potentials in isolated cardiomyocytes [86]. Another mice model study observed associations between short‐term exposures to UFP and decreased levels in isovolumic developed pressure and contractility compared with filtered air controls [87]. In addition, a controlled human study in both exercising heart‐failure patients and control patients found an association between increased levels of diesel exhaust with decreased left ventricular stroke volume (i.e., the amount of blood the left ventricle pumps per beat) [88]. These studies have confirmed that exposure to air pollution was sufficient to produce heart failure with anatomical evidence (dilated cardiomyopathy with ventricular volume changes, and ventricular wall thickening), functional measures (impaired pressure‐volume loops and deficits in contraction length), and cellular manifestation (delayed calcium reuptake during relaxation and reduced response to B‐adrenergic stimulation, increased cardiac collagen deposition) [85].

#### Stroke

Animal studies provide evidence for ANS pathway alterations and resulting stroke [9, 10]. Solid particles like PM can induce cerebral ischemia‐like phenotypes. Air pollutants caused features of stroke‐like neuronal loss and activation of microglia and astrocytes [89]. In rodent models, PM of nanoscale caused additive effects of air pollution and ischemic stroke on cerebral damage [90]. In vivo and in vitro models have provided evidence to suggest that air pollution would exacerbate stroke, with oxidative stress being an anticipated mechanism [91]. Oxidative stress was proposed to play a contributing role in the disruption of the blood–brain barrier after inhalation of vehicle exhausts [92, 93, 94]. Various types of PM can induce cellular‐molecular changes characteristic of the response to stroke (e.g., alterations in *N*‐methyl‐d‐aspartate channel activity, dopaminergic and glutamatergic disruption) and are associated with inflammation and oxidative stress [89]. Direct exposure of diesel exhaust particles (DEP) to isolated brain capillaries increased oxidative stress and inflammation and may alter blood–brain barrier permeability [92]. While there is debate as to whether inhaled particles can access the brain in sufficient numbers and penetrate throughout cerebral tissue, results from in vitro findings offer a means by which inhalation of (the smallest ultrafine) particles could confer increased susceptibility to the consequences of a stroke [95].

#### Arrhythmias, atrial fibrillation, and cardiac arrest

Animal and experimental studies have demonstrated multiple pathways by which air pollution can lead to arrhythmias, atrial fibrillation, and cardiac arrests such as ANS alterations, oxidative stress, and inflammation [9, 10]. Changes in the ANS contribute to abnormalities or worsening of arrhythmia measured in toxicological studies [96, 97]. ANS alteration may increase heart rate parameters and atrial pressure, which can trigger ventricular arrhythmias, and atrial fibrillation increases the risk of related CVD events such as heart failure and stroke [9, 10]. Alternatively, effects may be mediated through direct particle translocation across the blood–brain barrier, with the potential to modify autonomic nervous regulation and dysregulate heart rate, HRV, and blood pressure [10, 78]. A human autopsy study demonstrated the presence of combustion‐derived magnetite particles in both the brain and the heart but identifying the precise contribution of pollutant translocation is still very difficult [98]. Pulmonary instillation of DEP in rats increased the incidence of cardiac arrhythmia and the extent of MI [99].

#### Other suggested pathways

Recent studies suggest that air pollution may affect HPA activation and may constitute an important pathway for CVD risk [69]. Hypothalamic activation may increase brown adipose dysfunction, white adipose inflammation, and insulin resistance, whereas adrenal axis activation may increase glucocorticoids [100, 101]. Other pathways could be related to epigenomic changes. Some studies suggested novel associations between long‐term ambient air pollution exposure and site‐specific DNA methylation in purified monocytes. Inconsistent evidence on long‐term NO_2_ exposure and genome‐wide DNA methylation needs to be studied further. Human observational studies have suggested that PM exposure may determine loss of methylation in blood DNA, potentially reflecting activation of proinflammatory states in blood leukocytes [102, 103, 104].

Collectively, animal, experimental, and epidemiological studies provide evidence for CVD effects of PM and NO_x_ exposure through a number of known pathways for CVD development and death.

### Geographic differences and vulnerable population

Results from our umbrella review are very similar to what was observed in the GBD in 2019, where it was noted that Asia had higher effect estimates for CVD mortality and hypertension among the global population [105]. Notably, countries with higher air pollution levels like India and China also have higher absolute numbers for DALYs compared to countries with lower pollution levels [106]. It was also noted that DALYs attributable to CVDs and ambient air pollution decreased among high‐income countries compared to other countries. The dose‐response function is steeper among more polluted areas, but this relationship seems to be nonlinear, and there is no lower concentration threshold that can be considered safe for the population [107]. Overall, the source and composition of pollutants vary vastly among and within countries; thus, an assessment of the impact of air pollution on CVD in any given exposure setting is dependent on using dose‐response functions relevant to the context of the population exposure [69].

In this umbrella review, we observed that vulnerable population groups like the elderly, cardiac patients, and adults with higher body mass index are at increased risk for air pollution–related cardiovascular health effects. The elderly population may be more susceptible as a function of the gradual decline of biological and physiological processes of the aging process, reducing their resilience to air pollution exposure. Populations with pre‐existing cardiovascular conditions may be predisposed to a higher risk for exacerbations of underlying disease processes. People with a higher body mass index may have a higher risk of cardiovascular events following ambient air pollution exposure, due to their altered state of blood coagulability and metabolism. We were unable to draw conclusions from this umbrella review on other potentially vulnerable groups such as those of different ethnicity, socioeconomic status, and gender differences mostly based on inadequate data availability.

### Limitations and strength

The general strength of this umbrella review is that it provides a comprehensive overview of the effect of ambient air pollution on multiple cardiovascular outcomes. We were able to include a wide range of air pollutants and CVD outcomes, and we focused both on short‐ and long‐term exposures. We further harmonized all the effect estimates across the different meta‐analyses to increase comparability. However, some of the limitations should be considered. First, high heterogeneity was observed among the different meta‐analyses, which may weaken arguments for causality, although almost all estimates showed significant effects in the same direction. Second, the results of the umbrella review are limited to the evidence summarized in the meta‐analyses, which are prone to publication bias. However, most meta‐analyses did not find publication bias, but they cannot be fully ruled out. Third, we only included published systematic reviews and meta‐analyses until January 2021 and thus the most recent primary studies are likely not included in these reviews. This is especially relevant for new developing fields such as atrial fibrillation. Fourth, the quality of the meta‐analyses varied considerably with different search approaches and different effect estimates obtained from different study designs. However, we evaluated the quality of the reviews and most of them had an acceptable or high quality.

### Research gaps and future research

Although there is increasing evidence to support that air pollution has an important effect on cardiovascular health, there are still gaps that must be addressed in future research studies. Most of the evidence is collected for PM_2.5_ while other size fractions and components of PM are less well understood and would benefit from more research since toxicity might differ, as well as their source emissions. More research on multipollutant models, although methodologically challenging due to collinearity, might disentangle individual pollutant effects as well as explore the potential interaction between pollutants. This would better inform targeted interventions. From our review, we also observed that there is a need for more research on outcomes like heart failure, atherosclerosis, and specific arrhythmias such as atrial fibrillation, as well as more studies on NO_x_. Air quality should attend to safeguarding the health of vulnerable populations and research identifying these subpopulations is important to inform effective environmental health policy. In addition, it is clear that CVD effects of high levels of air pollution in LMICs warrant further research especially since these countries carry an enormous share of the burden of disease, have different source emissions than high‐income countries, and also potentially include other vulnerable populations based on housing conditions, concomitant diseases, nutritional status, and, for example, the additional burden of indoor air pollution from biomass burning. In the wake of the global COVID‐19 pandemic with large numbers of individuals with long‐term sequelae and possible lung injury and effects on immunity, we posit that it will be important to follow up on the potential consequences of air pollution exposure in these populations. Finally, specific air pollution mitigation programs are urgently needed to tackle adverse cardiovascular events, focusing on extremely polluted areas and vulnerable populations [5, 9, 10, 108, 109] and innovative designs evaluating the efficacy of interventions in reducing levels of air pollution and their effect on clinical CVD are warranted.

## Conclusion

This comprehensive umbrella review provides strong evidence that increased levels of ambient air pollution increase the risk of CVDs and CVD mortality, especially from stroke and IHD. The large and consistent evidence of these reviews emphasizes the importance of developing interventions to reduce the alarming levels of air pollution across the globe, especially in Asia and among vulnerable subpopulations.

## Conflict of interest

There is no conflict of interest to declare.

## Funding

This review was funded by an honorarium from JIM. J. D. B. was supported by funding from the Swedish Research Council for Sustainable Development FORMAS, S. J. was supported by India GEOHealth Hub (U01TW010097), and P. L. was supported by the Strategic Research Area Epidemiology at Karolinska Institutet.

## Author contributions

Jeroen de Bont: conceptualization; data curation; formal analyses; methodology; visualization; validation; writing – original draft (co‐lead); writing – review and editing (equal). Suganthi Jaganathan: conceptualization; data curation; formal analyses; methodology; visualization; validation; writing – original draft (co‐lead); writing – review and editing (equal). Marcus Dahlquist: conceptualization; writing – review and editing (equal). Åsa Persson: conceptualization; writing – review and editing (equal). Massimo Stafoggia: conceptualization; writing – review and editing (equal). Petter Ljungman: conceptualization; supervision; validation; writing – review and editing (equal).

## Supporting information


**Table S1**. Preferred Reporting Items for Systematic Review and Meta‐analysis (PRISMA‐P) guidelines of 2020.
**Table S2**: Search strategy in PubMed, EMBASE and Web of Science (WOS).
**Table S3**: Descriptive information of the included reviews.
**Table S4**. Meta‐analytic estimates: short‐term exposures to air pollution on adverse cardiovascular outcomes (effect estimates are specified as % increase in RR/HR/OR or beta (linear) for 10 µg/m^3^ increase of air pollution).
**Table S5**. Meta‐analytic estimates: long‐term exposures to air pollution on adverse cardiovascular outcomes (effect estimates are specified as % increase in RR/HR/OR or beta (linear) for 10 µg/m3 increase of air pollution).
**Table S6**. Systematic review results: short‐term exposures to air pollution and adverse cardiovascular outcomes.
**Table S7**. Systematic review results: long‐term exposures to air pollution and adverse cardiovascular outcomes.
**Figure S1**: Effect estimates of the association between short‐term exposure to PM_10_ and cardiovascular outcomes.
**Figure S2**: Effect estimates of the association between long‐term exposure to PM_10_ and cardiovascular outcomes.
