# Supplementary material for: Ambient air pollution and cardiovascular diseases: An umbrella review of systematic reviews and meta‐analyses
Source: J Intern Med. 2022 Mar 8;291(6):779–800. doi: 10.1111/joim.13467 (PMC9310863; doi:10.1111/joim.13467)
Supplement: Supplementary file 1 — Table S1. Preferred Reporting Items for Systematic Review and Meta‐analysis (PRISMA‐P) guidelines of 2020. Table S2: Search strategy in PubMed, EMBASE and Web of Science (WOS). Table S3: Descriptive information of the included reviews. Table S4. Meta‐analytic estimates: short‐term exposures to air pollution on adverse cardiovascular outcomes (effect estimates are specified as % increase in RR/HR/OR or beta (linear) for 10 µg/m3 increase of air pollution). Table S5. Meta‐analytic estimates: long‐term exposures to air pollution on adverse cardiovascular outcomes (effect estimates are specified as % increase in RR/HR/OR or beta (linear) for 10 µg/m3 increase of air pollution). Table S6. Systematic review results: short‐term exposures to air pollution and adverse cardiovascular outcomes. Table S7. Systematic review results: long‐term exposures to air pollution and adverse cardiovascular outcomes. Figure S1: Effect estimates of the association between short‐term exposure to PM10 and cardiovascular outcomes. Figure S2: Effect estimates of the association between long‐term exposure to PM10 and cardiovascular outcomes. [file JOIM-291-779-s001.docx]

**Supplementary material**

**Ambient air pollution and cardiovascular diseases: an umbrella review of systematic reviews and meta-analyses**

Jeroen de Bont^1#^, Suganthi Jaganathan^1,2,3#^, Marcus Dahlquist^1^, Åsa Persson^1^, Massimo Stafoggia^1,4^ and Petter Ljungman^1,5*^

^1^ Institute of Environmental Medicine, Karolinska Institutet, Sweden

^2^ Centre for Environmental Health, Public Health Foundation of India, Delhi-NCR, India

^3^ Centre for Chronic Disease Control, New Delhi, India

^4^ Department of Epidemiology, Lazio Region Health Service, ASL Roma 1, Italy

^5^ Department of Cardiology, Danderyd University Hospital, Sweden

# Shared first authorship

* Corresponding author: Institute of Environmental Medicine, Karolinska Institutet, SE-171 77, Stockholm, Sweden. E-mail address: petter.ljungman@ki.se

**Table S1.** Preferred Reporting Items for Systematic Review and Meta-analysis (PRISMA-P) guidelines of 2020.

**Table S2:** Search strategy in PubMed, EMBASE and Web of Science (WOS).

**Table S3:** Descriptive information of the included reviews.

**Table S4**. Meta-analytic estimates: short-term exposures to air pollution on adverse cardiovascular outcomes (effect estimates are specified as % increase in RR/HR/OR or beta (linear) for 10 μg/m^3^ increase of air pollution).

**Table S5**. Meta-analytic estimates: long-term exposures to air pollution on adverse cardiovascular outcomes (effect estimates are specified as % increase in RR/HR/OR or beta (linear) for 10 μg/m^3^ increase of air pollution).

**Table S6.** Systematic review results: short-term exposures to air pollution and adverse cardiovascular outcomes.

**Table S7.** Systematic review results: long-term exposures to air pollution and adverse cardiovascular outcomes.

**Figure S1:** Effect estimates of the association between short-term exposure to PM_10_ and cardiovascular outcomes.

**Figure S2:** Effect estimates of the association between long-term exposure to PM_10_ and cardiovascular outcomes.

**Table S1.** Preferred Reporting Items for Systematic Review and Meta-analysis (PRISMA-P) guidelines of 2020.

| **Section and Topic** | **Item #** | **Checklist item** | **Location where item is reported** |
| --- | --- | --- | --- |
| **TITLE** | | |  |
| Title | 1 | Identify the report as a systematic review. | 1 |
| **ABSTRACT** | | |  |
| Abstract | 2 | See the PRISMA 2020 for Abstracts checklist. | 2 |
| **INTRODUCTION** | | |  |
| Rationale | 3 | Describe the rationale for the review in the context of existing knowledge. | 2-3 |
| Objectives | 4 | Provide an explicit statement of the objective(s) or question(s) the review addresses. | 2-3 |
| **METHODS** | | |  |
| Eligibility criteria | 5 | Specify the inclusion and exclusion criteria for the review and how studies were grouped for the syntheses. | 3-4 |
| Information sources | 6 | Specify all databases, registers, websites, organisations, reference lists and other sources searched or consulted to identify studies. Specify the date when each source was last searched or consulted. | 3 |
| Search strategy | 7 | Present the full search strategies for all databases, registers and websites, including any filters and limits used. | 3/table S2 |
| Selection process | 8 | Specify the methods used to decide whether a study met the inclusion criteria of the review, including how many reviewers screened each record and each report retrieved, whether they worked independently, and if applicable, details of automation tools used in the process. | 3-4 |
| Data collection process | 9 | Specify the methods used to collect data from reports, including how many reviewers collected data from each report, whether they worked independently, any processes for obtaining or confirming data from study investigators, and if applicable, details of automation tools used in the process. | 3-4 |
| Data items | 10a | List and define all outcomes for which data were sought. Specify whether all results that were compatible with each outcome domain in each study were sought (e.g., for all measures, time points, analyses), and if not, the methods used to decide which results to collect. | 3-4 |
|  | 10b | List and define all other variables for which data were sought (e.g., participant and intervention characteristics, funding sources). Describe any assumptions made about any missing or unclear information. | 3-4 |
| Study risk of bias assessment | 11 | Specify the methods used to assess risk of bias in the included studies, including details of the tool(s) used, how many reviewers assessed each study and whether they worked independently, and if applicable, details of automation tools used in the process. | 3-4 |
| Effect measures | 12 | Specify for each outcome the effect measure(s) (e.g., risk ratio, mean difference) used in the synthesis or presentation of results. | 3-4 |
| Synthesis methods | 13a | Describe the processes used to decide which studies were eligible for each synthesis (e.g., tabulating the study intervention characteristics and comparing against the planned groups for each synthesis (item #5)). | 3-4 |
|  | 13b | Describe any methods required to prepare the data for presentation or synthesis, such as handling of missing summary statistics, or data conversions. | 3-4 |
|  | 13c | Describe any methods used to tabulate or visually display results of individual studies and syntheses. | 3-4 |
|  | 13d | Describe any methods used to synthesize results and provide a rationale for the choice(s). If meta-analysis was performed, describe the model(s), method(s) to identify the presence and extent of statistical heterogeneity, and software package(s) used. | 3-4 |
|  | 13e | Describe any methods used to explore possible causes of heterogeneity among study results (e.g., subgroup analysis, meta-regression). | NA |
|  | 13f | Describe any sensitivity analyses conducted to assess robustness of the synthesized results. | NA |
| Reporting bias assessment | 14 | Describe any methods used to assess risk of bias due to missing results in a synthesis (arising from reporting biases). | NA |
| Certainty assessment | 15 | Describe any methods used to assess certainty (or confidence) in the body of evidence for an outcome. | 3-4 |
| **RESULTS** | | |  |
| Study selection | 16a | Describe the results of the search and selection process, from the number of records identified in the search to the number of studies included in the review, ideally using a flow diagram. | 5 |
|  | 16b | Cite studies that might appear to meet the inclusion criteria, but which were excluded, and explain why they were excluded. | 5 |
| Study characteristics | 17 | Cite each included study and present its characteristics. | 5-9/Table S3 |
| Risk of bias in studies | 18 | Present assessments of risk of bias for each included study. | NA |
| Results of individual studies | 19 | For all outcomes, present, for each study: (a) summary statistics for each group (where appropriate) and (b) an effect estimates and its precision (e.g., confidence/credible interval), ideally using structured tables or plots. | 5-9/Figure 3-6/ Figures S1-2 |
| Results of syntheses | 20a | For each synthesis, briefly summarise the characteristics and risk of bias among contributing studies. | NA |
|  | 20b | Present results of all statistical syntheses conducted. If meta-analysis was done, present for each the summary estimate and its precision (e.g., confidence/credible interval) and measures of statistical heterogeneity. If comparing groups, describe the direction of the effect. | NA |
|  | 20c | Present results of all investigations of possible causes of heterogeneity among study results. | NA |
|  | 20d | Present results of all sensitivity analyses conducted to assess the robustness of the synthesized results. | NA |
| Reporting biases | 21 | Present assessments of risk of bias due to missing results (arising from reporting biases) for each synthesis assessed. | NA |
| Certainty of evidence | 22 | Present assessments of certainty (or confidence) in the body of evidence for each outcome assessed. | 8 |
| **DISCUSSION** | | |  |
| Discussion | 23a | Provide a general interpretation of the results in the context of other evidence. | 9 |
|  | 23b | Discuss any limitations of the evidence included in the review. | 13 |
|  | 23c | Discuss any limitations of the review processes used. | 13 |
|  | 23d | Discuss implications of the results for practice, policy, and future research. | 13-14 |
| **OTHER INFORMATION** | | |  |
| Registration and protocol | 24a | Provide registration information for the review, including register name and registration number, or state that the review was not registered. | 3 |
|  | 24b | Indicate where the review protocol can be accessed, or state that a protocol was not prepared. | 3 |
|  | 24c | Describe and explain any amendments to information provided at registration or in the protocol. | NA |
| Support | 25 | Describe sources of financial or non-financial support for the review, and the role of the funders or sponsors in the review. | 12 |
| Competing interests | 26 | Declare any competing interests of review authors. | 14 |
| Availability of data, code and other materials | 27 | Report which of the following are publicly available and where they can be found template data collection forms; data extracted from included studies; data used for all analyses; analytic code; any other materials used in the review. | Data extracted from included studies |

**Table S2:** Search strategy in PubMed, EMBASE and Web of Science (WOS).

| Database | Search strategy | Hits |
| --- | --- | --- |
| PUBMED | (Air Pollution[MeSH Terms] OR air pollut*[tiab] OR Traffic-Related Pollution[MeSH Terms] OR traffic* pollut*[tiab] OR vehicle pollut*[tiab] OR Vehicle Emissions[MeSH Terms] or vehic* emission*[tiab] or exhaust*[tiab]) AND (Particulate Matter[MeSH Terms] OR particulate matter[tiab] OR ultrafine fiber*[tiab] or ultrafine particle*[tiab] OR Nitrogen Oxides[MeSH Terms] OR nitrogen oxide*[tiab] OR nitrogen dioxide*[tiab] OR nitrous oxide*[tiab] OR dust[tiab] OR soot[tiab]) AND (Blood Pressure[MeSH Terms] OR blood pressure[tiab] OR Cardiovascular Diseases[MeSH Terms] OR cardiovascular disease*[tiab] OR arteriosclerosis[tiab] OR atherosclerosis[tiab] OR atrial fibrillation[tiab] OR cardiac arrhythmia*[tiab] OR cardiomyopath*[tiab] OR cerebrovascular disorder*[tiab] OR heart arrest[tiab] OR heart failure*[tiab] OR hypertension[tiab] OR myocardial infarction[tiab] OR myocardial ischemia[tiab] OR out-of-hospital cardiac arrest[tiab] OR stroke[tiab] OR Vascular Stiffness[MeSH Terms] OR vascular stiffness[tiab] OR Carotid Intima-Media Thickness[MeSH Terms] OR carotid intima-media thickness[tiab]) AND (meta-analysis[ti] OR meta-analysis[pt] OR review[ti] OR review[pt] OR systematic review[ti] OR systematic review[pt]) | 385 |
| EMBASE | ('air pollution':ab,ti OR 'traffic* pollut*':ab,ti OR 'vehicle pollut*':ab,ti OR 'vehic* emission*':ab,ti OR 'exhaust*':ab,ti) AND ('particulate matter':ab,ti OR 'ultrafine fiber*':ab,ti OR 'ultrafine particle*':ab,ti OR 'nitrogen oxide*':ab,ti OR 'nitrogen dioxide*':ab,ti OR 'nitrous oxide*':ab,ti OR 'dust':ab,ti OR 'soot':ab,ti) AND ('blood pressure':ab,ti OR 'cardiovascular disease*':ab,ti OR 'arteriosclerosis':ab,ti OR 'atherosclerosis':ab,ti OR 'atrial fibrillation':ab,ti OR 'cardiac arrhythmia*':ab,ti OR 'cardiomyopath*':ab,ti OR 'cerebrovascular disorder*':ab,ti OR 'heart arrest':ab,ti OR 'heart failure*':ab,ti OR 'hypertension':ab,ti OR 'myocardial infarction':ab,ti OR 'myocardial ischemia':ab,ti OR 'out-of-hospital cardiac arrest':ab,ti OR 'stroke':ab,ti OR 'vascular stiffness':ab,ti OR 'carotid intima-media thickness':ab,ti) AND ('meta-analysis':ab,ti OR 'meta analysis'/de OR 'systematic review':ab,ti OR 'systematic review'/de OR 'review':ab,ti OR review/it) | 225 |
| Web of science | ((air pollution) or (traffic* pollut*) or (vehicle pollut*) or (vehic* emission*) or (exhaust*)) and ((particulate matter) or (ultrafine fiber*) or (ultrafine particle*) or (nitrogen oxide*) or (nitrogen dioxide*) or (nitrous oxide*) or (dust) or (soot)) and ((blood pressure) or (cardiovascular disease*) or (arteriosclerosis) or (atherosclerosis) or (atrial fibrillation) or (cardiac arrhythmia*) or (cardiomyopath*) or (cerebrovascular disorder*) or (heart arrest) or (heart failure*) or (hypertension) or (myocardial infarction) or (myocardial ischemia) or (out-of-hospital cardiac arrest) or (stroke) or (vascular stiffness) or (carotid intima-media thickness)) and ((meta-analysis) or (systematic review) or (review)) | 502 |

Note: Search strategy was limited between 01/01/2010 and 31/01/2021 and reviews in English. Abbreviations: tiab or ab,ti, title and abstract; ti, title; pt or it, publication type

**Table S3.** Descriptive information of the included reviews.

| **Reference** | **Review type** | **Number of studies** | **Countries origin of studies of CVD studies** | **Study designs of CVD outcomes** | **AMSTAR** |
| --- | --- | --- | --- | --- | --- |
| Akintoye et al. 2016 [1] | SR & MA | 8 | US (4), Germany (3), Netherlands (1) | Cross-sectional (11), Longitudinal (4) | 4 out of 6 |
| Alexeeff et al. 2021 [2] | SR & MA | 69 (42 in meta-analysis) | US (31), Canada (13), England (5), China (4), Europe (4), Italy (3), Sweden (3), Australia (1), Switzerland (1), Germany (1), Korea (1), Israel (1), Taiwan (1) | Cohort (69) | 6 out of 7 |
| Atkinson et al. 2014 [3] | SR & MA | 110 | America (72), Europe (30), Western pacific (20), South-East Asia (1) | Time-series (110) | 6 out of 7 |
| Atkinson et al. 2015 [4] | SR & MA | 61 (related to 40 CVD) | North America (60), Europe (16), West Pacific (16), South America (4) | Time-series (61) | 4 out of 6 |
| Atkinson et al. 2018 [5] | SR & MA | 46 (22 related to CVD and 15 were included for meta-analysis) | Europe (13), North America (10), China (2), Japan (2), Taiwan (1) | Cohort (15) | 4 out of 7 |
| Burgan et al. 2010 [6] | SR | 49 | NA | Epidemiological studies (24), Experimental (25) | 2 out of 6 |
| Cai et al. 2016a [7] | SR & MA | 17 | China (3), Canada (3), US (2), Spain (2), Brazil (1), Iran (1), Germany (1), Taiwan (1), Sweden (1), Denmark (1) | Case-crossover (5), Cross-sectional (5), Cohort (4), Case-control (2), Time-series (1) | 4 out of 7 |
| Cai et al. 2016b [8] | SR & MA | 33 | US (21), Europe (3), England (3), Sweden (2), Taiwan (2), Canada (2), Brazil (2), China (1), France (1), Japan (1), Italy (1), Australia (1), Spain (1) | Case-crossover (9), Time-series (14) | 5 out of 6 |
| Chen et al. 2020 [9] | SR & MA | 107 (66 related to CVD outcomes) | US (26), Canada (11), China (6), Netherlands (3), UK (3), Italy (2), Israel (2), South Korea (2), Norway (2), Sweden (2), Europe (1), France (1), New Zealand (1), Switzerland (1), Taiwan (1), Japan (1) | Cohort (104), Case-control (3) | 6 out of 6 |
| Fajersztajn et al. 2017 [10] | SR & MA | 9 (3 related to CVD) | Chile (2), Mexico (1) | Time-series (3) | 6 out of 7 |
| Farhadi et al. 2020 [11] | SR & MA | 26 | US (8), Sweden (4), Germany (2), Canada (2), China (2), Spain (2), Australia (1), Japan (1), Italy (1), Taiwan (1), Finland (1), UK (1), Iran (1), Belgium (1) | Case-crossover (21), Time-series (7) | 7 out of 7 |
| Faustini et al. 2014 [12] | SR & MA | 23 studies (16 included for meta-analysis to CVD) | US (4), Canada (2), Sweden (2), Germany (2), Japan (2), Italy (1), China (1), New Zealand (1), Norway (1), France (1), Netherlands (1), | Cohort (14), Case-control (2) | 4 out of 7 |
| Fu et al. 2018 [13] | SR & MA | 80 | US (33), China (10), Canada (8), UK (5), Taiwan (5), Europe (3), Japan (3), Germany (2), Spain (2), Italy (1), Netherlands (1), Italy (1), Finland (1), Australia (1), Chile (1), LMIC (1), France (1) | Cohort (27), Case-crossover (26), Time- series (16), Cross-sectional (8), Case-control (3) | 6 out of 6 |
| Hoek et al. 2013 [14] | SR & MA | 39 (30 related to CVD) | US (12), Canada (3), Germany (2), Sweden (2), Italy (2), China (2), Japan (2), Netherlands (1), Switzerland (1), New Zealand (1), France (1), Scotland (1), Denmark (1) | Cohort (30) | 2 out of 7 |
| Jaganathan et al. 2019 [15] | SR | 17 | China (11), Brazil (3), Bulgaria (1), India (1), and Mexico (1) | Time-series (8), Cross-sectional (7), Time-stratified case-crossover (1), Cohort (1) | 3 out of 6 |
| Jilani et al. 2020 [16] | SR | 18 | US (7), Germany (2), Switzerland (1), Canada (1), Denmark (1), China (1), Australia (1), Netherlands (1), United Kingdom (1), Taiwan (1), Sweden (1) | Cross-sectional (13), Cohort (5) | 4 out of 6 |
| Kirrane et al. 2021 [17] | SR | 53 | US (34), Canada (6), China (4), Germany (4), South-Korea (2), Taiwan (1), Spain (1), Belgium (1) | Panel (30), Case crossover (9), Longitudinal (4), Cross-sectional (4), intervention (1), Repeated measures on prescribed exposures (3) | 5 out of 6 |
| Lai et al. 2013 [18] | SR & MA | 48 | China (48, including Hong Kong and Taiwan) | Not specified | 4 out of 6 |
| Li et al.2012 [19] | SR & MA | 12 | US (3), Taiwan (3), Europe (1), France (1), Denmark (1), Italy (1), South-Korea (1), Canada (1) | Time-series (7), Case-crossover (5) | 4 out of 7 |
| Liang et al. 2014 [20] | SR & MA | 22 | America (9), China (5), Canada (3) European Union (2); Taiwan (2), Belgium (1) | Panel (16), Cross-sectional (6) | 6 out of 6 |
| Liu et al. 2015 [21] | SR & MA | 11 | US (6), Canada (1), Taiwan (1), Europe (1), UK (1), Netherlands (1) | Cross-sectional (9), Longitudinal (1), Both designs (1) | 4 out of 7 |
| Liu et al. 2018 [22] | SR & MA | 16 | US (4), Netherlands (2), Canada (2), China (2), Japan (2), Italy (1), New Zealand (1), UK (1), Europe (1) | Cohort (16) | 4 out of 7 |
| Luben et al. 2017 [23] | SR | 25 | US (17), China (3), Europe (2), Canada (2), Netherlands (1), Taiwan (1), South-Korea (1) | Time-series (18), cohort (5), case-crossover (4), | 4 out of 6 |
| Luo et al. 2015 [24] | SR & MA | 31 | US (9), Taiwan (3), Sweden (2), Germany (2), France (2), Italy (2), Brazil (2), Canada (1), Australia (1), China (1), Netherlands (1), Europe (1), Spain (1), UK (1), Finland (1), Japan (1) | Case-crossover (19), Time-series (12) | 4 out of 7 |
| Ma et al. 2021 [25] | SR & MA | 11 | China (4), USA (6). Canada (1) | Cohort (6), Cross-sectional (5) | 3 out of 6 |
| Magalhaes et al. 2018 [26] | SR | 30 (28 related to outdoor air pollution and blood pressure) | US (15), Canada (5), China (3), Spain (2), Belgium (1), Korea (1), Taiwan (1), EU (1), India (1), Denmark (1) | Panel (26), Cross-sectional (9), Experimental (1) | 2 out of 6 |
| Mills et al. 2015 [27] | SR & MA | 204 (136 related to CVD) | Overall (CVD not specified): Europe (97), America (59), Western Pacific (59), Southeast Asia (2) | Time-series (136) | 5 out of 7 |
| Mills et al. 2016 [28] | SR & MA | 60 (28 related to CVD outcomes) | Not specified | Time-series (28) | 5 out of 6 |
| Mustafic et al. 2012 [29] | SR & MA | 34 | US (12), France (4), Italy (3), UK (2), Japan (2), Taiwan (2), Brazil (2), Australia/NZ (1), Sweden (1), Netherlands (1), Europe (1), Spain (1), Canada (1), Germany (1) | Time-series (17), Case-crossover (17) | 6 out of 7 |
| Newell et al. 2018 [30] | SR & MA | 60 (51 related to CVD) | China (33), Brazil (8), East Asia and pacific (7), Multi-city studies in Latin America and Caribbean (3), East Asia and china (2), Vietnam (1), Iran (2), Argentina (1), Turkey (1), South-Africa (1), Serbia (1), Malaysia (1), NA (1) | Time-series (53), Case-crossover (5), both (2) | 7 out of 7 |
| Niu et al. 2021 [31] | SR & MA | 68 | China (29), US (10), Taiwan (5), Canada (4), Japan (2), Brazil (2), South Korea (2), England (2), Sweden (2), Spain (1), Belgium (1), LIMC (1), Hong Kong (1), Germany (1), EU (1), Iceland (1), Denmark (1), Finland (1), France (1) | Time-series (28), Case-crossover (27), Cohort (13) | 4 out of 6 |
| Pranata et al. 2019 [32] | SR & MA | 49 | US (14), Canada (6), China (6), Taiwan (3), UK (3), South Korea (3), Sweden (2), Italy (2), Netherlands (2), Europe (2), Denmark (1), LMIC (1), Greece (1), Switzerland (1), Denmark (1), Japan (1) | Cohort (49) | 4 out of 6 |
| Provost et al. 2015 [33] | SR & MA | 9 | US (4), Germany (1), Netherlands (1), UK (1), Europe (1), Canada (1) | Cross-sectional (6 studies), Longitudinal (3 studies) | 4 out of 7 |
| Sangkharat et al. 2019 [34] | SR & MA | 33 | Japan (7), Australia (5), US (5), China (4), Lithuania (2), Spain (1), Germany (1), France (1), Austria (1), Italy (1), France (1), Sweden (1), Finland (1), Finland (1), Denmark, South-Korea (1) | Case-crossover (20), Time-series (12), both study designs (1) | 6 out of 6 |
| Scheers et al. 2014 [35] | SR & MA | 20 | US (5), China (3), UK (3), Canada (2), Japan (2), Europe (2), Netherlands (1), Switzerland (1), Greece (1) | Cohort (14), Ecological studies (6) | 4 out of 7 |
| Shah et al. 2013 [36] | SR & MA | 35 | USA (15), Canada (5), Italy (3), Taiwan (2), Japan (2), Australia & New Zealand (2), Hong Kong (2), Brazil (1), South Korea (1), UK (1), Netherlands (1) | Time-series (24), Case-crossover (10) | 4 out of 7 |
| Shah et al. 2015 [37] | SR & MA | 103 | US (21), China (17), Italy (11), Canada (6), Spain (5), France (5), Japan (5), Finland (4), UK (4), Taiwan (4), South Korea (3), Brazil (3), Australia (3), Europe Union (2), Chile (2), Netherlands (1), Denmark (1), Sweden (1), Russia (1), Thailand (1), Iran (1), Iceland (1), South Africa (1) | Time-series (69), Case-crossover (33), both study designs (1) | 6 out of 6 |
| Shao et al. 2015 [38] | SR & MA | 4 | US (2), UK (1), Canada (1) | Case-crossover (4) | 4 out of 7 |
| Shin et al. 2014 [39] | SR & MA | 20 | North America (13), Asia (2), Europe (3). Australia (1), England (1) | Cohort (4), Time-series/Case-crossover (15), Multi-stage modelling (1) | 3 out of 6 |
| Song et al. 2016 [40] | SR & MA | 25 | USA (7), Canada (4), Taiwan (3), China (2), Italy (2), UK (2), Australia (1), Brazil (1), Japan (1), Netherlands (1), Finland (1) | Time-series (13), Case-crossover (11); both study designs (1) | 6 out of 6 |
| Stieb et al. 2020 [41] | SR & MA | 86 (67 included in the meta-analysis) | US (13), China (12), Canada (8), UK (6), Taiwan (6), France (6), Japan (4), Sweden (4), Europe (3), Belgium (3), Iran (3), Australia (2), Finland (2), Italy (2), Poland (2), Spain (1), Germany (1), Lithuania(1), Denmark (1), New Zealand and Australia (1), Chile (1), South-Korea (1), Thailand (2),Brazil (1), Hong-Kong and UK (1) | Time-series (48), Case-crossover (38) | 7 out of 7 |
| Teng et al. 2013 [42] | SR | 8 | USA (5), Denmark (1), Finland (1), Australia (1) | Case-crossover (8) | 3 out of 6 |
| Wang et al. 2014 [43] | SR & MA | 45 | US (9), China (6), Italy (4), Canada (3), UK (3), Taiwan (3), France (3), Spain (3), Europe (1), Denmark (1), Brazil (1), Sweden (1), Finland (2), Australia (1), Japan (1), Korea (2), Russia (1), Latin America (1) | Times-series (28), Case-crossover (17) | 4 out of 7 |
| Yamamoto et al. 2014 [44] | SR | 9 (3 related to outdoor air pollution and related to our search strategy) | India (3) | Cross-sectional (2), Time-series (1) | 2 out of 6 |
| Yang et al. 2014 [45] | SR & MA | 34 | China (8), US (7), Japan (3), Canada (4), UK (2), European Union (2), Finland (2), South-Korea (2), France (1), Iceland (1), Denmark (1), Spain (1) | Time-series (20), Case-crossover (14) | 6 out of 7 |
| Yang et al. 2018 [46] | SR & MA | 65 articles (100 studies) | China (23), US (20), Canada (8), Brazil (3), South Korea (2), Belgium (2), Europe (2), Netherlands (1), Sweden (1), Germany (1), Denmark (1), France (1) | Panel (26), Cross-sectional (16), Cohort (15), Case-crossover (7), Cross-sectional/cohorts (1) | 6 out of 7 |
| Yang et al. 2019 [47] | SR & MA | 35 | US (7), China (6), Canada (4), UK (4), Italy (2), Netherlands (2), Europe (2), France (2), Japan (2), Sweden (2), Norway (1), Switzerland (1), Greece (1), Korea (1), LMIC (1) | Cohort (35) | 5 out of 6 |
| Yu et al. 2014 [48] | SR & MA | 19 | China (6), US (4), UK (2), Canada (2), Australia (1), Denmark (1), Finland (1), France (1), Italy (1) | Time-series (11), Case-crossover (8) | 4 out of 7 |
| Yuan et al. 2019 [49] | SR & MA | 16 | US (5), Europe (2), China (2), Netherlands (1), Multiple LMIC countries (1), South-Korea (1), UK (1), Sweden (1), Italy (1) | Cohort (16) | 7 out of 7 |
| Yue et al. 2020 [50] | SR & MA | 4 | US (3), China (1) | Case-crossover (4) | 4 out of 7 |
| Yue et al. 2021 [51] | SR & MA | 18 | China (6), Sweden (2), Korea (2), US (2), Italy (2), Canada (1), Iran (1), Israel (1), Denmark (1) | Time-series (8), Case-crossover (5), Cohort (5) | 4 out of 7 |
| Zanoli et al. 2017 [52] | SR | 8 | NA | NA | 2 out of 6 |
| Zhao et al. 2017 [53] | SR & MA | 15 | US (5), Australia (2), Denmark (1), France (1), Finland (1), Sweden (1), Japan (1); China (2), South-Korea (1) | Case-crossover (11), Time-series (2), both (2) | 6 out of 7 |
| Zhang et al. 2020 [54] | SR | 31 (not specified) | China (31) | Not specified | 1 out of 6 |
| Zhao et al. 2017 [55] | SR & MA | 30 | China (30) | Time-series (28), Case-crossover (2) | 6 out of 7 |
| Zhu et al. 2021 [56] | SR & MA | 12 | Canada (3), US (2), Denmark (1), Netherlands (1), LMIC (1), China (1), South Korea (1), Israel (1), UK (1) | Cohort (12) | 4 out of 7 |

**Abbreviations:** MA, meta-analysis; SR, systematic review

**Table S4**. Meta-analytic estimates: short-term exposures to air pollution on adverse cardiovascular outcomes (effect estimates are specified as % increase in RR/HR/OR or beta (linear) for 10 μg/m^3^ increase of air pollution).

| **Air pollutant** | **Outcome** | **Risk estimates** | **Effect estimates**  **(95% CI)** | **Heterogeneity I^2^ (%)** | **Reference** |
| --- | --- | --- | --- | --- | --- |
| **1.All cause CVD mortality & morbidity** | | | | | |
|  | Mortality | Excess RR | 0.84 (0.41, 1.28) | 76% | Atkinson et al. 2014 [3] |
|  | Mortality | Excess RR | 1.00 (1.00, 2.00) | 0% | Fajersztajn et al. 2017 [10] |
|  | Mortality | Excess RR | 0.68 (0.39, 0.97) | 90% | Zhao et al. 2017 [53] |
|  | Hospital admission | Excess RR | 0.90 (0.26, 1.53) | 0% | Atkinson et al. 2014 [3] |
| PM_2.5_ | Hospital admissions, 65+ years | Excess RR | 1.78 (0.48, 3.10) | 85% | Atkinson et al. 2014 [3] |
|  | Ambulance dispatch | Excess RR | 0.00 (-2.00, 2.00) | 73% | Sangkharat et al. 2019 [34] |
|  | Ambulance dispatch | Excess RR | 4.00 (-2.00, 10.00) | 85% | Sangkharat et al. 2019 [34] |
|  | Physician diagnosis | Excess RR | 0.00 (-1.00, 1.00) | 0% | Sangkharat et al. 2019 [34] |
| PM_10_ | Mortality | Excess RR | 0.49 (0.35, 0.63) | 61% | Lai et al. 2013 [18] |
|  | Mortality | Excess RR | 0.39 (0.26, 0.53) | 83% | Zhao et al. 2017 [53] |
|  | Hospital admission | Excess RR | 0.210 (0.01, 0.41) |  | Lai et al. 2013 [18] |
|  | Emergency room visit | Excess RR | 0.300 (0.01, 0.600) |  | Lai et al. 2013 [18] |
|  | Emergency admission | Excess RR | 0.600 (0.09, 1.11) |  | Lai et al. 2013 [18] |
|  | Ambulance dispatch | Excess RR | 1.00 (0.00, 2.00) | 51% | Sangkharat et al. 2019 [34] |
| PM | Mortality | Excess RR | 0.48 (0.18, 0.78) | 67% | Mills et al. 2016 [28] |
| SPM | Physician diagnosis | Excess RR | 1.00 (-1.00, 2.00) | 71% | Sangkharat et al. 2019 [34] |
| EC | Mortality | Excess Risk | 17.90 (5.35, 31.93) | 97% | Atkinson et al. 2015 [4] |
| PM-nitrate | Mortality | Excess Risk | 1.11 (-1.29, 3.56) | 70% | Atkinson et al. 2015 [4] |
| PM-organic carbon | Mortality | Excess Risk | 5.74 (0.23, 11.56) | 97% | Atkinson et al. 2015 [4] |
| PM-sulphate | Mortality | Excess Risk | 2.12 (-0.20, 4.49) | 42% | Atkinson et al. 2015 [4] |
| NO_2_/NO_x_ | Mortality | Excess RR | 1.62 (1.18, 2.06) |  | Lai et al. 2013 [18] |
|  | Mortality | Excess RR | 0.88 (0.63, 1.13) | 72% | Mills et al. 2015 [27] |
|  | Mortality | Excess RR | 1.07 (0.43, 1.72) | 72% | Mills et al. 2016 [28] |
|  | Mortality | Excess RR | 0.92 (0.44, 1.39) | 83% | Newell et al. 2018 [30] |
|  | Mortality | Excess RR | 1.12 (0.76, 1.48) | 67% | Zhao et al. 2017 [53] |
|  | Hospital admission | Excess RR | 0.95 (0.54, 1.37) |  | Lai et al. 2013 [18] |
|  | Hospital admission | Excess RR | 0.66 (0.32, 1.01) | 88% | Mills et al. 2015 [27] |
|  | Hospital admission | Excess RR | 0.93 (0.46, 1.40) |  | Mills et al. 2016 [28] |
|  | Hospital admission | Excess RR | 0.79 (-0.08, 1.66) | 51% | Newell et al. 2018 [30] |
|  | Emergency room visit | Excess RR | 1.40 (-0.001, 2.82) |  | Lai et al. 2013 [18] |
| NO_2_/NO_x_ | Emergency admission | Excess RR | 1.30 (0.59, 2.02) |  | Lai et al. 2013 [18] |
| **2.Ischemic heart disease & Myocardial Infarction** | | | | | |
| PM_2.5_ | IHD mortality | Excess RR | 3.36 (0.68, 6.10) | 90% | Atkinson et al. 2014 [3] |
|  | IHD hospital admission, 65+ years | Excess RR | 2.52 (0.53, 4.55) | 86% | Atkinson et al. 2014 [3] |
|  | MI mortality/hospital admission | Excess RR | 2.50 (1.50, 3.60) | 51% | Mustafic et al. 2012 [29] |
|  | MI mortality | Excess RR | 1.20 (1.00, 1.50) | 62% | Cai et al. 2016b [8] |
|  | MI hospital admission | Excess RR | 2.40 (0.70, 4.10) | 85% | Cai et al. 2016b [8] |
|  | MI hospital admission | Excess RR | 2.00 (1.00,3.00) | 70% | Farhadi et al. 2020 [11] |
|  | MI hospital admission | Excess OR | 2.20 (1.50, 3.00) | 61% | Luo et al. 2015 [24] |
| PM_10_ | IHD mortality | Excess RR | -0.37 (1.38, 0.65) |  | Lai et al. 2013 [18] |
|  | IHD hospital admission | Excess RR | 0.65 (0.26, 1.05) |  | Lai et al. 2013 [18] |
|  | IHD emergency admission | Excess RR | 0.70 (-0.11, 1.51) |  | Lai et al. 2013 [18] |
|  | IHD emergency room visit | Excess RR | 0.10 (-0.80, 1.01) |  | Lai et al. 2013 [18] |
|  | MI mortality/hospital admission | Excess RR | 0.60 (0.20, 0.90) | 57% | Mustafic et al. 2012 [29] |
|  | MI mortality | Excess RR | 0.80 (0.40, 1.20) | 52% | Cai et al. 2016b [8] |
|  | MI hospital admission | Excess RR | 1.10 (0.60, 1.60) | 67% | Cai et al. 2016b [8] |
|  | MI hospital admission | Excess OR | 0.50 (0.10, 0.80) | 56% | Luo et al. 2015 [24] |
| NO_2_/NO_x_ | IHD mortality | Excess RR | 1.61 (0.24, 1.97) | 50% | Mills et al. 2015 [27] |
|  | IHD mortality | Excess RR | 1.30 (-1.02, 3.67) |  | Lai et al. 2013 [18] |
|  | IHD hospital admission | Excess RR | 0.88 (0.52, 1.20) | 0% | Mills et al. 2015 [27] |
|  | IHD hospital admission | Excess RR | 1.42 (-0.04, 2.90) |  | Lai et al. 2013 [18] |
|  | IHD hospital admission (case-crossover) | Excess RR | 3.87 (2.71, 5.05) | 91% | Stieb et al. 2020 [41] |
|  | IHD hospital admission (time-series) | Excess RR | 1.16 (0.80, 1.53) | 95% | Stieb et al. 2020 [41] |
|  | IHD emergency admission | Excess RR | 1.00 (-0.01, 2.02) |  | Lai et al. 2013 [18] |
|  | MI mortality/hospital admission | Excess RR | 1.10 (0.60, 1.60) | 71% | Mustafic et al. 2012 [29] |
| **4.Blood pressure & Hypertension** | | | | | |
| PM_2.5_ | BP systolic | Beta increase of mmHg | 1.09 (0.62 1.57) | 80% | Liang et al. 2014 [20] |
|  | BP systolic | Beta increase of mmHg | 0.53 (0.26, 0.80) | 71% | Yang et al. 2018 [46] |
|  | BP systolic* | Beta increase of mmHg | 1.39 (0.87, 1.91) | 84% | Liang et al. 2014 [20] |
|  | BP diastolic | Beta increase of mmHg | 0.52 (0.25, 0.79) | 70% | Liang et al. 2014 [20] |
|  | BP diastolic | Beta increase of mmHg | 0.20 (0.02, 0.38) | 65% | Yang et al. 2018 [46] |
|  | BP diastolic* | Beta increase of mmHg | 0.90 (0.49, 1.30) | 88% | Liang et al. 2014 [20] |
|  | Hypertension | Excess OR | 6.90 (0.30, 14.10) | 78% | Cai et al. 2016a [7] |
| PM_2.5_ | Hypertension | Excess OR | 10.00 (6.00, 13.00) | 0% | Yang et al. 2018 [46] |
| PM_10_ | BP systolic | Beta increase of mmHg | 0.21 (-0.01, 0.43) | 90% | Yang et al. 2018 [46] |
|  | BP diastolic | Beta increase of mmHg | 0.15 (0.01, 0.29) | 88% | Yang et al. 2018 [46] |
|  | Hypertension | Excess OR | 2.40 (1.60, 3.20) | 21% | Cai et al. 2016a [7] |
|  | Hypertension | Excess OR | 6.00 (2.00, 10.00) | 85% | Yang et al. 2018 [46] |
|  | Hypertension emergency room visit | Excess RR | 1.88 (0.51, 3.27) |  | Lai et al. 2013 [18] |
| NO_2_/NO_x_ | BP systolic | Beta increase of mmHg | 0.23 (-0.19, 0.64) | 93% | Yang et al. 2018 [46] |
|  | BP diastolic | Beta increase of mmHg | 0.28 (0.15, 0.41) | 76% | Yang et al. 2018 [46] |
|  | Hypertension | Excess OR | 3.70 (-0.60, 8.20) | 81% | Cai et al. 2016a [7] |
|  | Hypertension | Excess OR | 5.00 (2.00, 8.00) | 57% | Yang et al. 2018 [46] |
|  | Hypertension emergency room visit | Excess RR | 6.89 (1.06, 13.05) |  | Lai et al. 2013 [18] |
| **5.Heart failure** | | | | | |
| PM_2.5_ | Mortality/hospital admission | Excess RR | 2.12 (1.42, 2.82) | 53% | Shah et al. 2013 [36] |
|  | hospital admission | Excess RR | 0.39 (1.35, 7.53) | 65% | Atkinson et al. 2014 [3] |
| PM_10_ | Mortality/hospital admission | Excess RR | 1.63 (1.20, 2.07) | 75% | Shah et al. 2013 [36] |
|  | Emergency room visit | Excess RR | 1.20 (-0.71, 3.14) |  | Lai et al. 2013 [18] |
| NO_2_/NO_x_ | Mortality/hospital admission | Excess RR | 0.90 (0.66, 1.14) | 91% | Shah et al. 2013 [36] |
|  | Hospital admission | Excess RR | 1.41 (-0.01, 2.86) | 85% | Mills et al. 2015 [27] |
| **6.Stroke** | | | | | |
| PM_2.5_ | Mortality/hospital admission | Excess RR | 1.20 (0.22, 2.18) | 59% | Yang et al. 2014 [45] |
|  | Mortality | Excess RR | 1.85 (0.74, 2.97) | 50% | Atkinson et al. 2014 [3] |
|  | Mortality | Excess OR | 2.00 (1.00, 4.00) | 69% | Fu et al. 2018 [13] |
|  | Mortality | Excess RR | 1.20 (1.10, 1.20) | 92% | Shah et al 2015 [37] |
|  | Mortality | Excess RR | 1.34 (0.27, 2.42) |  | Yang et al. 2014 [45] |
|  | Mortality* | Excess OR | 3.00 (2.00, 4.00) | 64% | Fu et al. 2018 [13] |
|  | Mortality* | Excess HR | 0.80 (0.50, 1.20) | 89% | Niu et al. 2021 [31] |
|  | Hospital admission | Excess OR | 0.60 (0.20, 1.00) | 49% | Li et al. 2012 [19] |
|  | Hospital admission | Excess HR | 0.80 (0.50, 1.10) | 97% | Niu et al. 2021 [31] |
|  | Hospital admission | Excess RR | 1.10 (1.00, 1.20) | 85% | Shah et al. 2015 [37] |
|  | Hospital admission | Excess RR | 0.70 (0.30, 1.00) |  | Shin et al. 2014 [39] |
|  | Hospital admission | Excess RR | 0.50 (−0.19, 2.93) |  | Yang et al. 2014 [45] |
|  | Hospital admission | Excess OR | -0.10 (-0.60, 0.30) | 52% | Yu et al. 2014 [48] |
|  | Hospital admission, 65+ years | Excess RR | -0.45 (-2.21, 1.33) | 79% | Atkinson et al. 2014 [3] |
| PM_2.5_ | Incidence | Excess OR | 1.00 (1.00, 2.00) | 94% | Fu et al. 2018 [13] |
|  | Incidence* | Excess OR | 2.00 (1.00, 2.00) | 93% | Fu et al. 2018 [13] |
|  | Incidence* | Excess HR | 4.80 (2.00, 7.60) | 82% | Niu et al. 2021 [31] |
|  | CeVD mortality | Excess RR | 1.40 (0.90, 1.90) | 7% | Wang et al. 2014 [43] |
|  | CeVD hospital admission | Excess RR | 0.60 (0.20, 1.00) |  | Shin et al. 2014 [39] |
|  | CeVD hospital admission | Excess RR | 0.30 (-0.50, 1.20) | 64% | Wang et al. 2014 [43] |
| PM_2.5_ | Haemorrhagic stroke mortality/hospital admission | Excess RR | 1.22 (−0.55, 3.02) |  | Yang et al. 2014 [45] |
|  | Haemorrhagic stroke hospital admission | Excess RR | 0.40 (-2.20, 2.90) | 14% | Shah et al. 2015 [37] |
|  | Haemorrhagic stroke hospital admission | Excess RR | 1.20 (-8.00, 11.00) |  | Shin et al. 2014 [39] |
|  | Haemorrhagic stroke hospital admission | Excess RR | -1.00 (-4.50, 2.60) | 0% | Wang et al. 2014 [43] |
|  | Haemorrhagic stroke hospital admission | Excess OR | -3.20 (-5.90, -0.50) |  | Yu et al. 2014 [48] |
|  | Ischemic stroke mortality/hospital admission | Excess RR | 1.04 (−0.25, 2.34) |  | Yang et al. 2014 [45] |
|  | Ischemic stroke hospital admission | Excess RR | 1.00 (0.80, 1.10) | 69% | Shah et al. 2015 [37] |
|  | Ischemic stroke hospital admission | Excess RR | 4.00 (1.00, 7.00) |  | Shin et al. 2014 [39] |
|  | Ischemic stroke hospital admission | Excess RR | 1.30 (-4.20, 7.00) | 64% | Wang et al. 2014 [43] |
|  | Ischemic stroke hospital admission | Excess OR | 2.50 (0.10, 4.90) | 46% | Yu et al. 2014 [48] |
| PM_10_ | Mortality | Excess RR | 0.62 (0.33, 0.92) |  | Lai et al. 2013 [18] |
|  | Mortality | Excess RR | 0.30 (0.20, 0.40) | 25% | Shah et al. 2015 [37] |
|  | Mortality | Excess RR | 0.65 (0.54, 0.77) |  | Yang et al. 2014 [45] |
|  | Mortality* | Excess HR | 0.60 (0.30, 1.00) | 83% | Niu et al. 2021 [31] |
|  | Hospital admission | Excess OR | 0.20 (-0.10, 0.50) | 61% | Li et al.2012 [19] |
|  | Hospital admission | Excess HR | 0.40 (0.10, 0.60) | 93% | Niu et al. 2021 [31] |
|  | Hospital admission | Excess RR | 0.20 (0.00, 0.30) | 20% | Shah et al. 2015 [37] |
|  | Hospital admission | Excess RR | 0.71 (0.10, 1.33) |  | Yang et al. 2014 [45] |
|  | Hospital admission | Excess OR | 0.40 (0.10, 0.80) | 70% | Yu et al. 2014 [48] |
|  | Incidence* | Excess HR | 1.70 (-1.90, 5.50) | 52% | Niu et al. 2021 [31] |
|  | Mortality/hospital admission | Excess RR | 0.58 (0.31, 0.86) | 67% | Yang et al. 2014 [45] |
|  | CeVD mortality | Excess RR | 0.57 (0.22, 0.93) |  | Lai et al. 2013 [18] |
|  | CeVD mortality | Excess RR | 0.50 (0.30, 0.70) | 84% | Wang et al. 2014 [43] |
|  | CeVD hospital admission | Excess RR | -0.19 (-0.97, 0.60) |  | Lai et al. 2013 [18] |
| PM_10_ | CeVD hospital admission | Excess RR | 0.30 (-0.10, 0.80) | 77% | Wang et al. 2014 [43] |
|  | CeVD emergency room visit | Excess RR | 0.10 (-0.51, 0.71) |  | Lai et al. 2013 [18] |
|  | CeVD emergency admission | Excess RR | 0.30 (-0.41, 1.01) |  | Lai et al. 2013 [18] |
|  | Haemorrhagic stroke mortality/hospital admission | Excess RR | 0.68 (−0.91, 2.29) |  | Yang et al. 2014 [45] |
|  | Haemorrhagic stroke hospital admission | Excess RR | 0.20 (-0.30, 0.60) | 0% | Shah et al. 2015 [37] |
|  | Haemorrhagic stroke hospital admission | Excess RR | 0.90 (-2.40, 4.30) | 79% | Wang et al. 2014 [43] |
|  | Haemorrhagic stroke hospital admission | Excess OR | 0.70 (0.80, 2.20) | 55% | Yu et al. 2014 [48] |
|  | Ischemic stroke mortality/hospital admission | Excess RR | 0.72 (−0.06, 1.50) |  | Yang et al. 2014 [45] |
|  | Ischemic stroke hospital admission | Excess RR | 0.20 (-0.10, 0.40) | 23% | Shah et al. 2015 [37] |
|  | Ischemic stroke hospital admission | Excess RR | 0.00 (-2.40, 2.40) | 87% | Wang et al. 2014 [43] |
|  | Ischemic stroke hospital admission | Excess OR | 1.30 (0.10, 2.50) | 73% | Yu et al. 2014 [48] |
| PM_coarse_ | CeVD mortality | Excess RR | 0.70 (-0.50, 1.90) | 46% | Wang et al. 2014 [43] |
|  | CeVD hospital admission | Excess RR | -1.00 (-4.50, 3.80) | 80% | Wang et al. 2014 [43] |
| NO_2_/NO_x_ | Mortality/hospital admission | Excess RR | 1.19 (0.62, 1.76) | 83% | Yang et al. 2014 [45] |
|  | Mortality | Excess RR | 1.35 (0.74, 2.99) | 0% | Mills et al. 2015 [27] |
|  | Mortality | Excess RR | 1.76 (0.68, 2.85) | 26% | Mills et al. 2016 [28] |
|  | Mortality | Excess RR | 1.01 (0.73, 1.43) | 28% | Newell et. al. 2018 [30] |
|  | Mortality | Excess RR | 0.80 (0.20, 1.39) |  | Yang et al. 2014 [45] |
|  | Mortality | Excess RR | 0.85 (0.48, 1.22) | 22% | Shah et al. 2015 [37] |
|  | Mortality* | Excess HR | 0.90 (0.30, 1.60) | 70% | Niu et al. 2021 [31] |
|  | Hospital admission | Excess RR | 0.30 (0.02, 0.57) | 0% | Mills et al. 2015 [27] |
|  | Hospital admission | Excess RR | 1.08 (0.73, 1.43) | 86% | Newell et al. 2018 [30] |
|  | Hospital admission | Excess HR | 2.30 (1.50, 3.00) | 93% | Niu et al. 2021 [31] |
|  | Hospital admission | Excess RR | 0.64 (0.32, 0.95) | 61% | Shah et al. 2015 [37] |
|  | Hospital admission | Excess RR | 1.89 (0.85, 2.94) |  | Yang et al. 2014 [45] |
|  | Incidence* | Excess HR | 0.20 (0.00, 0.30) | 0% | Niu et al. 2021 [31] |
|  | CeVD mortality | Excess RR | 1.47 (0.70, 2.25) |  | Lai et al. 2013 [18] |
|  | CeVD hospital admission | Excess RR | 0.32 (-0.09, 0.73) |  | Lai et al. 2013 [18] |
|  | CeVD emergency admission | Excess RR | 0.80 (-0.20, 1.81) |  | Lai et al. 2013 [18] |
|  | Haemorrhagic stroke mortality/hospital admission | Excess RR | 0.62 (-0.43, 1.67) |  | Yang et al. 2014 [45] |
| NO_2_/NO_x_ | Haemorrhagic stroke hospital admission | Excess RR | 1.27 (0.18, 2.37) | 42% | Shah et al. 2015 [37] |
|  | Ischemic stroke mortality/hospital admission | Excess RR | 1.74 (0.69, 2.81) |  | Yang et al. 2014 [45] |
|  | Ischemic stroke hospital admission | Excess RR | 1.27 (0.54, 2.00) | 56% | Shah et al. 2015 [37] |
| **7.Arrhythmias, Atrial Fibrillation, Cardiac Arrest** | | | | | |
| PM_2.5_ | Arrhythmia mortality | Excess RR | 2.70 (-1.30, 6.80) | 57% | Song et al. 2016 [40] |
|  | Arrhythmia hospital admission | Excess RR | 1.50 (0.50, 2.50) | 78% | Song et al. 2016 [40] |
|  | Arrhythmia hospital admission, 65+ years | Excess RR | 0.60 (0.03, 1.17) | 0% | Atkinson et al. 2014 [3] |
|  | Atrial fibrillation | Excess RR | 0.90 (0.20, 1.60) | 65% | Shao et al. 2015 [38] |
| PM_2.5_ | Atrial fibrillation | Excess OR | 1.00 (0.00, 2.00) | 71% | Yue et al. 2021 [51] |
|  | Atrial fibrillation - ICD patients | Excess OR | 24.00 (0.00, 53.00) | 73% | Yue et al. 2020 [50] |
|  | Cardiac arrest | Excess RR | 4.00 (1.00, 7.00) | 70% | Zhang et al. 2017 [54] |
|  | Cardiac arrest ambulance dispatch | Excess RR | 2.00 (-1.00, 5.00) | 28% | Sangkharat et al. 2019 [34] |
|  | Cardiac arrest paramedic assessment | Excess RR | 5.00 (3.00, 8.00) | 60% | Sangkharat et al. 2019 [34] |
| PM_10_ | Arrhythmia mortality | Excess RR | 0.90 (-0.60, 2.40) | 13% | Song et al. 2016 [40] |
|  | Arrhythmia hospital admission | Excess RR | 0.90 (0.40, 1.50) | 80% | Song et al. 2016 [40] |
|  | Arrhythmia emergency room visit | Excess RR | 0.20 (-0.80, 1.21) |  | Lai et al. 2013 [18] |
|  | Atrial fibrillation | Excess OR | 3.00 (1.00, 5.00) | 77% | Yue et al. 2021 [51] |
|  | Cardiac arrest | Excess RR | 2.00 (1.00, 4.00) | 78% | Zhang et al. 2017 [54] |
|  | Cardiac arrest paramedic | Excess RR | 2.00 (0.00, 5.00) | 69% | Sangkharat et al. 2019 [34] |
| PM_coarse_ | Cardiac arrest paramedic | Excess RR | 4.00 (1.00, 6.00) | 25% | Sangkharat et al. 2019 [34] |
| NO_2_/NO_x_ | Arrhythmia mortality | Excess RR | 1.38 (0.14, 2.63) | 24% | Song et al. 2016 [40] |
|  | Arrhythmia hospital admission | Excess RR | 1.90 (0.92, 2.89) | 94% | Song et al. 2016 [40] |
|  | Arrhythmia hospital admission | Excess RR | 0.50 (0.15, 0.86) | 0% | Mills et al. 2015 [27] |
|  | Atrial fibrillation | Excess RR | 0.53 (0.01, 1.06) | 0% | Shao et al. 2015 [38] |
|  | Atrial fibrillation | Excess OR | 1.59 (1.06, 2.11) | 7% | Yue et al. 2021 [51] |
|  | Cardiac arrest paramedic | Excess RR | 0.00 (-2.07, 2.11) | 67% | Sangkharat et al. 2019 [34] |
|  | Cardiac arrest | Excess RR | 2.00 (1.00, 3.00) | 66% | Zhang et al. 2017 [54] |

* Effect estimates are estimated for short-and long-term exposures combined, as reported by the study authors. **Abbreviations:** ABI, ankle-brachial index; BP, blood pressure; CAC, coronary artery calcium; CIMT, carotid intima-media thickness test; CVD, cardiovascular diseases; CeVD, cerebrovascular diseases; DBP, diastolic blood pressure; HR, hazard ratio; ICD, implantable cardioverter defibrillator; IHD, ischemic heart diseases; MA, meta-analyses; MI, myocardial infarction; NO_2_, nitrogen dioxide; NO_x_, nitrogen oxides; OR, odds ratio; PM_2.5_, particulate matter with an aerodynamic diameter of less than 2.5 μm; PM_10_, particulate matter with an aerodynamic diameter of less than 10μm; PM_coarse_, particulate matter with an aerodynamic diameter of between 2.5 than 10μm; RR, relative risk; SPM, suspended particulate matter; SBP, systolic blood pressure; SR, systematic review; TSP, total suspended particles; UFP, ultrafine particle

**Table S5**. Meta-analytic estimates: long-term exposures to air pollution on adverse cardiovascular outcomes (effect estimates are specified as % increase in RR/HR/OR or beta (linear) for 10 μg/m^3^ increase of air pollution).

| **Air pollutant** | **Outcome** | **Risk estimates** | **Effect estimates**  **(95% CI)** | **Heterogeneity I^2^ (%)** | **Reference** |
| --- | --- | --- | --- | --- | --- |
| **1.All cause CVD mortality & morbidity** | | | | | |
| PM_2.5_ | Mortality/incidence | Excess RR | 11.00 (7.00, 14.00) |  | Yang et al. 2019 [47] |
|  | Mortality | Excess RR | 14.00 (8.00, 21.00) | 99% | Alexeeff et al. 2021 [2] |
|  | Mortality | Excess RR | 11.00 (9.00, 14.00) | 72% | Chen et al. 2020 [9] |
|  | Mortality | Excess RR | 20.00 (9.00, 31.00) | 98% | Faustini et al. 2014 [12] |
|  | Mortality | Excess RR | 10.60 (5.40, 16.0) | 94% | Hoek et al. 2013 [14] |
|  | Mortality | Excess HR | 12.00 (8.00, 16.00) | 92% | Liu et al. 2018 [22] |
|  | Mortality | Excess HR | 10.00 (7.00, 12.00) | 85% | Pranata et al. 2019 [32] |
|  | Mortality | Excess RR | 13.00 (6.00, 20.00) | 61% | Yang et al. 2019 [47] |
|  | Incidence | Excess HR | 9.00 (1.00, 17.00) | 94% | Pranata et al. 2019 [32] |
|  | Incidence | Excess RR | 11.00 (7.00, 14.00) | 1% | Yang et al. 2019 [47] |
| PM_10_ | Mortality/incidence | Excess RR | 12.00 (-10.00, 33.00) | 83% | Yang et al. 2019 [47] |
|  | Mortality | Excess RR | 4.00 (-1.00, 10.00) | 99% | Chen et al. 2020 [9] |
|  | Mortality | Excess HR | 2.00 (-11.0, 16.00) | 95% | Liu et al. 2018 [22] |
|  | Mortality | Excess HR | 17.00 (4.00, 30.00) | 99% | Pranata et al. 2019 [32] |
|  | Mortality | Excess RR | 19.00 (9.00, 30.00) |  | Yang et al. 2019 [47] |
|  | Incidence | Excess HR | 23.00 (-11.00, 71.00) | 100% | Pranata et al. 2019 [32] |
|  | Incidence | Excess RR | 12.00 (5.00, 19.00) | 44% | Yang et al. 2019 [47] |
| NO_2_/NO_x_ | Mortality/incidence | Excess RR | 11.00 (7.00, 15.00) |  | Yang et al. 2019 [47] |
|  | Mortality | Excess HR | 3.00 (2.00, 5.00) | 83% | Atkinson et al. 2018 [5] |
|  | Mortality | Excess RR | 13.00 (9.00, 18.00) | 98% | Faustini et al. 2014 [12] |
|  | Mortality | Excess HR | 17.00 (10.00, 25.00) | 99% | Pranata et al. 2019 [32] |
|  | Mortality | Excess RR | 14.00 (8.00, 21.00) |  | Yang et al. 2019 [47] |
|  | Incidence | Excess HR | 15.00 (2.00, 29.00) | 99% | Pranata et al. 2019 [32] |
|  | Incidence | Excess RR | 12.00 (8.00, 16.00) |  | Yang et al. 2019 [47] |
| **2.Ischemic heart disease & Myocardial Infarction** | | | | | |
| PM_2.5_ | IHD mortality/incidence | Excess RR | 23.00 (15.00, 31.00) |  | Yang et al. 2019 [47] |
|  | IHD mortality/incidence | Excess HR | 4.00 (0.00, 9.00) | 39% | Pranata et al. 2019 [32] |
|  | IHD mortality/incidence | Excess HR | 15.00 (12.00, 17.00) | 43% | Pranata et al. 2019 [32] |
|  | IHD mortality | Excess RR | 23.00 (15.00, 31.00) | 94% | Alexeeff et al. 2021 [2] |
|  | IHD mortality | Excess RR | 16.00 (10.00, 21.00) | 78% | Chen et al. 2020 [9] |
| PM_2.5_ | IHD mortality | Excess HR | 11.00 (7.00, 16.00) | 76% | Pranata et al. 2019 [32] |
| PM_2.5_ | IHD mortality | Excess RR | 5.00 (2.00, 7.00) | 13% | Yang et al. 2019 [47] |
|  | IHD incidence | Excess RR | 1.00 (-1.00, 3.00) | 0% | Yang et al. 2019 [47] |
|  | MI mortality | Excess HR | 7.00 (4.00, 9.00) | 21% | Zhu et al. 2021 [56] |
|  | MI incidence | Excess HR | 10.00 (2.00, 18.00) | 68% | Zhu et al. 2021 [56] |
|  | MI incidence | Excess RR | 8.00 (-1.00, 18.00) | 84% | Alexeeff et al. 2021 [2] |
| PM_10_ | IHD mortality/incidence | Excess RR | 1.00 (-2.00, 4.00) | 0% | Yang et al. 2019 [47] |
|  | IHD mortality/incidence | Excess HR | 9.00 (-2.00, 21.00) | 78% | Pranata et al. 2019 [32] |
|  | IHD mortality/incidence | Excess HR | 20.00 (-15.00, 69.00) | 99% | Pranata et al. 2019 [32] |
|  | IHD mortality | Excess RR | 6.00 (1.00, 10.00) | 73% | Chen et al. 2020 [9] |
|  | IHD mortality | Excess HR | 3.00 (1.00, 5.00) | 0% | Pranata et al. 2019 [32] |
|  | IHD mortality | Excess RR | 12.00 (2.00, 22.00) | 0% | Yang et al. 2019 [47] |
|  | IHD incidence | Excess RR | 2.00 (-6.00, 10.00) | 52% | Yang et al. 2019 [47] |
| NO_2_/NO_x_ | IHD mortality/incidence | Excess RR | 17.00 (10.00, 23.00) |  | Yang et al. 2019 [47] |
|  | IHD mortality/incidence | Excess HR | 2.00 (-5.00, 10.00) | 76% | Pranata et al. 2019 [32] |
|  | IHD mortality/incidence | Excess HR | 8.00 (2.00, 13.00) | 85% | Pranata et al. 2019 [32] |
|  | IHD mortality | Excess HR | 5.00 (3.00, 8.00) | 73% | Pranata et al. 2019 [32] |
|  | IHD mortality | Excess RR | 5.00 (4.00, 6.00) |  | Yang et al. 2019 [47] |
|  | IHD incidence | Excess RR | 1.00 (-2.00, 3.00) |  | Yang et al. 2019 [47] |
| **3.Atherosclerosis & Arterial Stiffness** | | | | | |
| PM_2.5_ | CIMT | Excess RR | 22.52 (-1.26, 46.29) | 83% | Akintoye et al. 2016 [1] |
|  | CIMT | Beta (linear) | 16.79 μm (4.95, 28.63) | 70% | Liu et al. 2015 [21] |
|  | CIMT (cross-sectional) | Percent change in CIMT | 3.35 (1.74, 4.98) | 11% | Provost et al. 2015 [33] |
|  | CIMT (longitudinal) | Change in CIMT progression in um per year | 2.09 (0.04, 4.18) | 0% | Provost et al. 2015 [33] |
|  | CAC | Excess RR | 1.35 (0.62, 2.95) | 60% | Akintoye et al. 2016 [1] |
|  | ABI | Excess RR | 0.00 (-0.02, 0.01) | 0% | Akintoye et al. 2016 [1] |
| PM_10_ | CIMT | Beta (linear) | 4.13 μm (−5.79, 14.04) | 67% | Liu et al. 2015 [21] |
| **4.Blood pressure & Hypertension** | | | | | |
| PM_2.5_ | BP systolic | Beta increase of mmHg | 7.35 (-0.01 to 14.70) | 93% | Liang et al. 2014 [20] |
|  | BP systolic | Beta increase of mmHg | 0.37 (-0.65, 1.39) | 83% | Yang et al. 2018 [46] |
|  | BP diastolic | Beta increase of mmHg | 9.49 (-1.85, 20.83) | 98% | Liang et al. 2014 [20] |
| PM_2.5_ | BP diastolic | Beta increase of mmHg | 0.47 (0.12, 0.82) | 78% | Yang et al. 2018 [46] |
| PM_2.5_ | Hypertension | Excess OR | 6.90 (0.30, 14.10) | 68% | Cai et al. 2016a [7] |
|  | Hypertension | Excess HR | 7.00 (1.00, 14.00) | 98% | Pranata et al. 2019 [32] |
|  | Hypertension | Excess OR | 5.00 (1.00, 9.00) | 48% | Yang et al. 2018 [46] |
|  | Hypertension in woman | Excess OR | 7.00 (0.00, 14.00) | 69% | Ma et al. 2021 [25] |
|  | Hypertension in woman | Excess HR | 23.00 (8.00, 40.00) | 89% | Ma et al. 2021 [25] |
| PM_10_ | BP systolic | Beta increase of mmHg | 0.14 (-0.37, 0.66) | 95% | Yang et al. 2018 [46] |
|  | BP diastolic | Beta increase of mmHg | 0.86 (0.37, 1.35) | 87% | Yang et al. 2018 [46] |
|  | Hypertension | Excess OR | 4.00 (-1.00, 9.00) | 83% | Yang et al. 2018 [46] |
|  | Hypertension | Excess OR | 5.40 (3.60, 7.20) | 48% | Cai et al. 2016a [7] |
| PM_coarse_ | BP systolic | Excess OR | 5.00 (2.00, 8.00) | 57% | Yang et al. 2018 [46] |
|  | Hypertension | Excess OR | 1.00 (-6.00, 7.00) | 35% | Yang et al. 2018 [46] |
| NO_2_/NO_x_ | BP systolic | Beta increase of mmHg | -0.05 (-0.11, 0.02) | 32% | Yang et al. 2018 [46] |
|  | BP diastolic | Beta increase of mmHg | 0.08 (-0.05, 0.29) | 91% | Yang et al. 2018 [46] |
|  | Hypertension | Excess OR | 3.40 (0.50, 6.30) | 38% | Cai et al. 2016a [7] |
|  | Hypertension | Excess OR | 0.00 (-2.00, 1.00) | 61% | Yang et al. 2018 [46] |
|  | Hypertension | Excess OR | 12.70 (-6.70, 36.40) | 98% | Cai et al. 2016a [7] |
| **5.Heart failure** | | | | | |
| PM_2.5_ | Incidence | Excess HR | 7.00 (-28.00, 60.00) | 72% | Pranata et al. 2019 [32] |
| PM_10_ | Incidence | Excess HR | 25.00 (4.00, 50.00) | 82% | Pranata et al. 2019 [32] |
| NO_2_/NO_x_ | Incidence | Excess HR | 42.00 (-7.00, 118.00) | 97% | Pranata et al. 2019 [32] |
| **6.Stroke** | | | | | |
| PM_2.5_ | Mortality/incidence | Excess RR | 9.00 (-14.00, 29.00) |  | Yang et al. 2019 [47] |
|  | Mortality | Excess RR | 11.00 (4.00, 18.00) | 85% | Chen et al. 2020 [9] |
|  | Mortality | Excess OR | 15.00 (7.00, 24.00) | 42% | Fu et al. 2018 [13] |
|  | Mortality | Excess HR | 26.56 (1.54, 57.75) | 65% | Scheers et al. 2014 [35] |
|  | Mortality | Excess RR | 11.00 (-4.00, 26.00) | 93% | Yang et al. 2019 [47] |
|  | Mortality | Excess HR | 11.00 (5.00, 17.00) | 26% | Yuan et al. 2019 [49] |
|  | Incidence | Excess RR | 13.00 (11.00, 15.00) | 0% | Alexeeff et al. 2021 [2] |
|  | Incidence | Excess OR | 14.00 (8.00, 21.00) | 45% | Fu et al. 2018 [13] |
|  | Incidence | Excess HR | 13.00 (6.00, 19.00) | 73% | Pranata et al. 2019 [32] |
|  | Incidence | Excess HR | 13.21 (4.21, 22.99) | 60% | Scheers et al. 2014 [35] |
|  | Incidence | Excess RR | 8.00 (-14.00, 29.00) | 99% | Yang et al. 2019 [47] |
|  | Incidence | Excess HR | 11.0 (5.00, 17.0) | 77% | Yuan et al. 2019 [49] |
| PM_2.5_ | Hospital admission | Excess RR | 6.00 (0.00, 13.00) |  | Shin et al. 2014 [39] |
|  | CeVD mortality | Excess RR | 24.00 (13.00, 36.00) | 94% | Alexeeff et al. 2021 [2] |
| PM_10_ | Mortality/incidence | Excess RR | -3.00 (-17.00, 10.00) | 89% | Yang et al. 2019 [47] |
|  | Mortality | Excess RR | 1.00 (-0.17, 21.00) | 99% | Chen et al. 2020 [9] |
|  | Mortality | Excess HR | 9.10 (-0.42, 24.20) | 75% | Scheers et al. 2014 [35] |
|  | Mortality | Excess RR | -1.00 (-19.00, 18.00) | 23% | Yang et al. 2019 [47] |
|  | Incidence | Excess HR | 15.00 (-10.00, 47.00) | 99% | Pranata et al. 2019 [32] |
|  | Incidence | Excess HR | 2.10 (-2.50, 6.90) | 31% | Scheers et al. 2014 [35] |
|  | Incidence | Excess RR | -1.00 (-19.00, 18.00) | 82% | Yang et al. 2019 [47] |
| PM_10_ or (converted) PM_2.5_ exposure | Mortality | Excess HR | 8.00 (-0.80, 17.70) | 91% | Scheers et al. 2014 [35] |
|  | Incidence | Excess HR | 6.10 (1.80, 10.50) | 86% | Scheers et al. 2014 [35] |
| NO_2_/NO_x_ | Mortality/incidence | Excess RR | 6.00 (0.00, 12.00) |  | Yang et al. 2019 [47] |
|  | Mortality | Excess RR | 8.00 (-5.00, 20.00) |  | Yang et al. 2019 [47] |
|  | Incidence | Excess HR | 13.00 (0.00, 28.00) | 95% | Pranata et al. 2019 [32] |
|  | Incidence | Excess RR | 4.00 (-10.00, 17.00) |  | Yang et al. 2019 [47] |
| **7.Arrhythmias, Atrial Fibrillation, Cardiac Arrest** | | | | | |
| PM_2.5_ | Atrial fibrillation | Excess HR | -7.00 (-32.00, 27.00) | 80% | Pranata et al. 2019 [32] |
|  | Atrial fibrillation | Excess OR | 7.00 (4.00, 10.00) | 38% | Yue et al. 2021 [51] |
| PM_10_ | Atrial fibrillation | Excess HR | -9.00 (-26.00, 12.00) | 67% | Pranata et al. 2019 [32] |
| NO_2_/NO_x_ | Atrial fibrillation | Excess HR | 1.00 (1.00, 2.00) | 0% | Pranata et al. 2019 [32] |
|  | Atrial fibrillation | Excess OR | 1.06 (0.02, 2.11) | 73% | Yue et al. 2021 [51] |

**Abbreviations:** ABI, ankle-brachial index; BP, blood pressure; CAC, coronary artery calcium; CIMT, carotid intima-media thickness test; CVD, cardiovascular diseases; CeVD, cerebrovascular diseases; DBP, diastolic blood pressure; HR, hazard ratio; ICD, implantable cardioverter defibrillator; IHD, ischemic heart diseases; MA, meta-analyses; MI, myocardial infarction; NO_2_, nitrogen dioxide; NO_x_, nitrogen oxides; OR, odds ratio; PM_2.5_, particulate matter with an aerodynamic diameter of less than 2.5 μm; PM_10_, particulate matter with an aerodynamic diameter of less than 10μm; PM_coarse_, particulate matter with an aerodynamic diameter of between 2.5 than 10μm; RR, relative risk; SPM, suspended particulate matter; SBP, systolic blood pressure; SR, systematic review; TSP, total suspended particles; UFP, ultrafine particle.

**Table S6.** Systematic review results: short-term exposures to air pollution and adverse cardiovascular outcomes.

| **Air pollutant** | **Outcome** | **Statistically significant studies/included studies** | **Consistency of results** | **Reference** |
| --- | --- | --- | --- | --- |
| **1. All cause CVD mortality & morbidity** | | | | |
| PM_2.5_ | Mortality | 6 out of 8 | Consistent in direction but not in statistical significance | Luben et al. 2017 [23] |
|  | Mortality | 10 out of 10 | Consistent in direction and statistical significance | Zhang et al. 2020 [54] |
|  | Hospital admission | 4 out of 7 | Consistent in direction but not in statistical significance | Luben et al. 2017 [23] |
|  | Hospital admission | 4 out of 4 | Consistent in direction and positive association | Jaganathan et al. 2019 [15] |
| EC | Mortality | 3 out of 8 | Consistent in direction but not in statistical significance | Luben et al. 2017 [23] |
|  | Hospital admission | 7 out of 7 | Consistent in direction and statistical significance | Luben et al. 2017 [23] |
| **2. Ischemic heart disease & Myocardial Infarction** | | | | |
| PM_2.5_ | IHD mortality | 1 out of 1 | Only 1 study available | Luben et al. 2017 [23] |
|  | IHD mortality | 5 out of 5 | Consistent in direction and statistical significance | Zhang et al. 2020 [54] |
|  | IHD hospital admission | 1 out of 1 | Only 1 study available | Luben et al. 2017 [23] |
|  | MI mortality | 2 out of 2 | Only 2 studies available | Zhang et al. 2020 [54] |
|  | MI hospital admission | 2 out of 3 | Only 3 studies available | Zhang et al. 2020 [54] |
| PM | IHD hospital admission | 5 out of 8 | Consistent in direction but not in statistical significance | Burgan et al. 2010 [6] |
|  | MI hospital admission | 4 out of 5 | Consistent in direction and statistical significance | Burgan et al. 2010 [6] |
| EC | IHD mortality | 1 out of 1 | Only 1 study available | Luben et al. 2017 [23] |
|  | IHD hospital admission | 1 out of 1 | Only 1 study available | Luben et al. 2017 [23] |
| **3. Atherosclerosis & Arterial Stiffness** | | | | |
| PM | Arterial stiffness | 4 out of 5 | Consistent in direction and statistical significance | Zanoli et al. 2017 [52] |
| NO_2_/NO_x_ | Arterial stiffness | 1 out of 2 | Only 2 studies available | Zanoli et al. 2017 [52] |
| **4. Blood pressure & Hypertension** | | | | |
| PM_2.5_ | BP | Not specified | No consistent pattern according to the authors | Kirrane et al. 2021 [17] |
| BC | BP | Not specified | No consistent pattern according to the authors | Kirrane et al. 2021 [17] |
| EC/BC | BP systolic | 14 out of 19 | Consistent in direction and statistical significance | Magalhaes et al. 2018 [26] |
|  | BP diastolic | 14 out of 19 | Consistent in direction and statistical significance | Magalhaes et al. 2018 [26] |
| UFP | BP systolic | 9 out of 11 | Consistent in direction and statistical significance | Magalhaes et al. 2018 [26] |
|  | BP diastolic | 7 out of 11 | Consistent in direction but not in statistical significance | Magalhaes et al. 2018 [26] |
| **6. Stroke** | | | | |
| PM_2.5_ | Hospital admission | 1 out of 1 | Only 1 study available | Luben et al. 2017 [23] |
|  | CeVD mortality | 2 out of 2 | Only 2 studies available | Zhang et al. 2020 [54] |
|  | CeVD hospital admission | 10 out of 10 | Consistent in direction and statistical significance | Zhang et al. 2020 [54] |
| BC | Hospital admission | 1 out of 1 | Only 1 study available | Luben et al. 2017 [23] |
| **7. Arrhythmias, Atrial Fibrillation, Cardiac Arrest** | | | | |
| PM_2.5_ | Arrhythmia | 1 out of 8 | Consistent in direction but not in statistical significance | Kirrane et al. 2021 [17] |
|  | Cardiac arrest incidence | 5 out of 8 | Consistent in direction but not in statistical significance | Teng et al. 2013 [42] |
| PM_10_ | Cardiac arrest incidence | 4 out of 5 | Consistent in direction and statistical significance | Teng et al. 2013 [42] |
| PM | Arrhythmia | 1 out of 4 | Inconsistent, only 4 studies available | Burgan et al. 2010 [6] |
| BC | Arrhythmia | 2 out of 8 | Consistent in direction but not in statistical significance | Kirrane et al. 2021 [17] |
| NO_2_/NO_x_ | Cardiac arrest incidence | 1 out of 7 | Inconsistent | Teng et al. 2013 [42] |

**Abbreviations:**  BC, black carbon, BP, blood pressure; CVD, cardiovascular diseases; CeVD, cerebrovascular diseases; EC, elemental carbon; IHD, ischemic heart diseases; MI, myocardial infarction; NO_2_, nitrogen dioxide; NO_x_, nitrogen oxides; e matter with an aerodynamic diameter of less than 2.5 μm; PM_10_, particulate matter with an aerodynamic diameter of less than 10μm; PM_coarse_, particulate matter with an aerodynamic diameter of between 2.5 than 10μm; SPM, suspended particulate matter; UFP, ultrafine particle.

**Table S7.** Systematic review results: long-term exposures to air pollution and adverse cardiovascular outcomes.

| **Air pollutant** | **Outcome** | **Statistically significant studies/included studies** | **Consistency of results** | **Reference** |
| --- | --- | --- | --- | --- |
| **1. All cause CVD mortality & morbidity** | | | | |
| PM_2.5_ | Mortality | 8 out of 9 | Consistent in direction and in statistical significance | Jaganathan et al. 2019 [15] |
|  | Mortality | 1 out of 1 | Only 1 study available | Luben et al. 2017 [23] |
|  | Hospital admission | 1 out of 1 | Only 1 study available | Luben et al. 2017 [23] |
| SPM | Mortality | 1 out of 1 | Only 1 study available | Yamamoto et al. 2014 [44] |
| EC | Mortality | 2 out of 2 | Only 2 study available | Luben et al. 2017 [23] |
|  | Hospital admission | 1 out of 1 | Only 1 study available | Luben et al. 2017 [23] |
| NO_2_/NO_x_ | Mortality | 1 out of 1 | Only 1 study available | Yamamoto et al. 2014 [44] |
| **2. Ischemic heart disease & Myocardial Infarction** | | | | |
| PM_2.5_ | IHD mortality | 2 out of 2 | Only 2 studies available | Luben et al. 2017 [23] |
|  | MI hospital admission | 1 out of 1 | Only 1 study available | Zhang et al. 2020 [54] |
| EC | IHD mortality | 2 out of 2 | Only 2 studies available | Luben et al. 2017 [23] |
| **3. Atherosclerosis & Arterial Stiffness** | | | | |
| PM_2.5_ | CIMT | 1 out of 4 | Inconsistent | Jilani et al. 2020 [16] |
|  | CAC | 1 out of 2 | Only 2 studies available | Jilani et al. 2020 [16] |
| PM | CIMT | 6 out of 9 | Consistent in direction but not in statistical significance | Jilani et al. 2020 [16] |
|  | CAC | 4 out of 7 | Consistent in direction but not in statistical significance | Jilani et al. 2020 [16] |
| NO_2_/NO_x_ | Arterial stiffness | 1 out of 1 | Only 1 study available | Zanoli et al. 2017 [52] |
| **4. Blood pressure & Hypertension** | | | | |
| BC | BP | 3 out of 3 | Only 3 studies available | Magalhaes et al. 2018 [26] |
| PM_2.5_ | Hypertension | 1 out of 1 | Only 1 study available | Jaganathan et al. 2019 [15] |
| **6. Stroke** | | | | |
| PM_2.5_ | CeVD hospital admission | 2 out of 2 | Only two studies available | Zhang et al. 2020 [54] |

**Abbreviations:**  BC, black carbon, BP, blood pressure; CAC, coronary artery calcium; CIMT, carotid intima-media thickness test; CVD, cardiovascular diseases; CeVD, cerebrovascular diseases; EC, elemental carbon; IHD, ischemic heart diseases; MI, myocardial infarction; NO_2_, nitrogen dioxide; NO_x_, nitrogen oxides; e matter with an aerodynamic diameter of less than 2.5 μm; PM_10_, particulate matter with an aerodynamic diameter of less than 10μm; PM_coarse_, particulate matter with an aerodynamic diameter of between 2.5 than 10μm; SPM, suspended particulate matter.

**Figure S1:** Effect estimates of the association between short-term exposure to PM_10_ and cardiovascular outcomes.


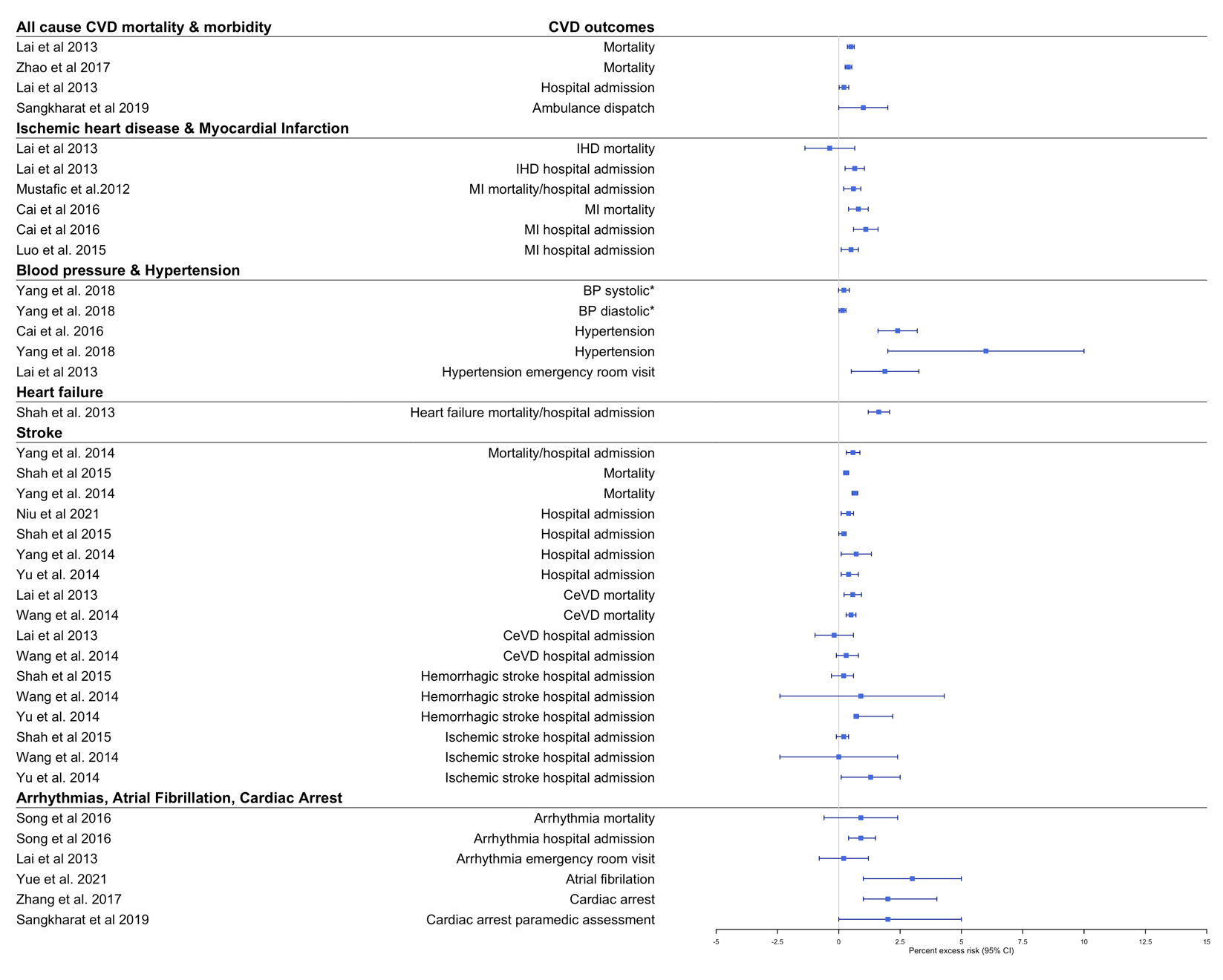


Note: we selected the main effect estimate from the meta-analyses if multiple effect estimates were available for each CVD outcome in the same meta-analysis. Effect estimates are estimated per 10 µg/m^3^ range increase in PM_10._ Abbreviations: BP, blood pressure; CeVD, cerebrovascular diseases; CVD, cardiovascular diseases; IHD, ischemic heart diseases; MI, myocardial infarction.

*Beta coefficient (linear regression) for change in systolic and diastolic values per increase of PM_10_

**Figure S2:** Effect estimates of the association between long-term exposure to PM_10_ and cardiovascular outcomes.


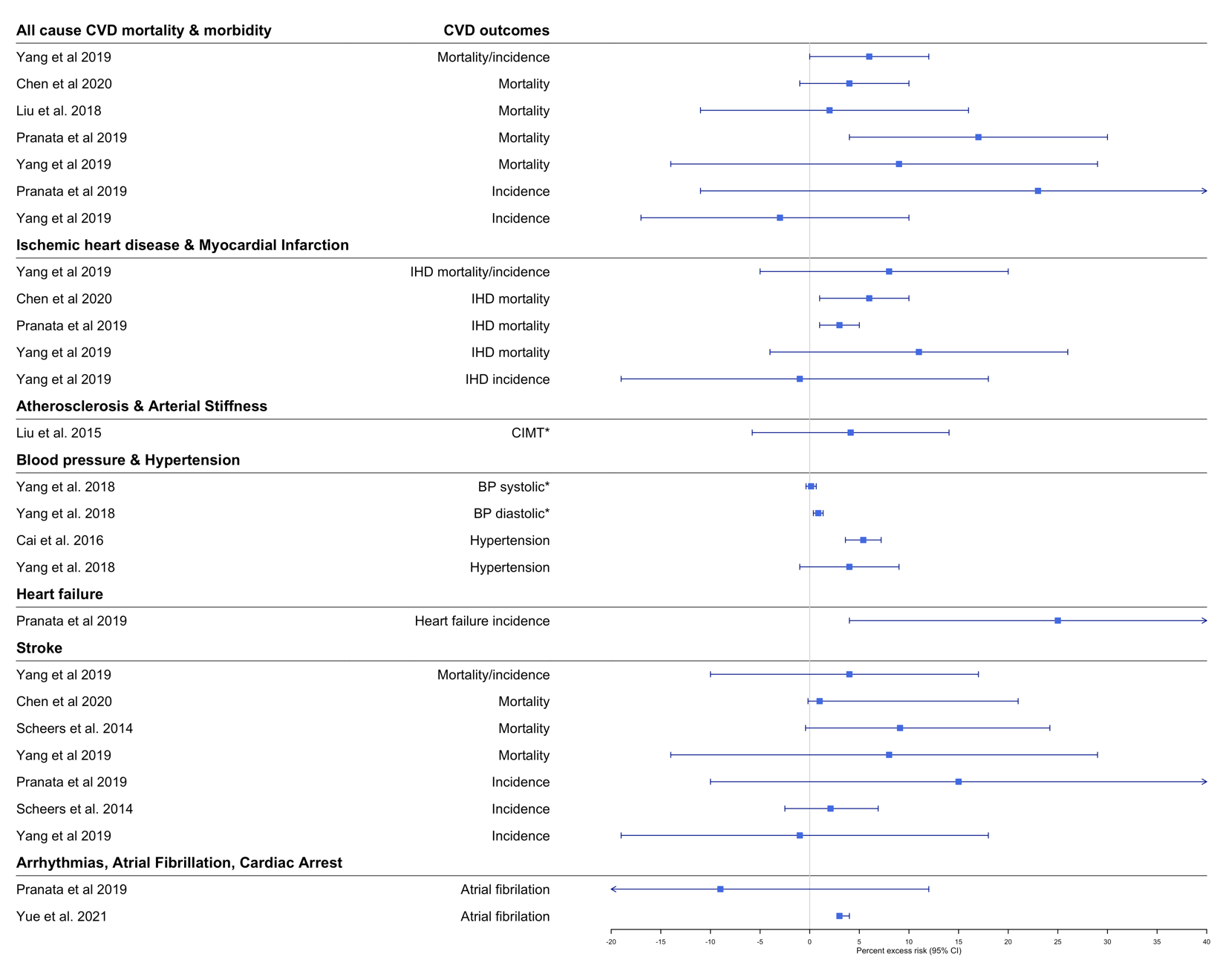


Note: we selected the main effect estimate from the meta-analyses if multiple effect estimates were available for each CVD outcome in the same meta-analysis. Effect estimates are estimated per 10 µg/m^3^ range increase in PM_10._ Abbreviations: BP, blood pressure; CeVD, cerebrovascular diseases; CIMT, carotid intima-media thickness test; CVD, cardiovascular diseases; IHD, ischemic heart diseases; MI, myocardial infarction.

*Beta coefficient (linear regression) for change in systolic, diastolic, or CIMT values per increase of PM_10_

**References:**

1 Akintoye E, Shi L, Obaitan I, Olusunmade M, Wang Y, Newman JD, Dodson JA. Association between fine particulate matter exposure and subclinical atherosclerosis: A meta-analysis. *Eur J Prev Cardiol* 2016; **23:** 602-12.

2 Alexeeff SE, Liao NS, Liu X, Van Den Eeden SK, Sidney S. Long-Term PM2.5  Exposure and Risks of Ischemic Heart Disease and Stroke Events: Review and Meta-Analysis. *J Am Heart Assoc* 2021; **10:** e016890.

3 Atkinson RW, Kang S, Anderson HR, Mills IC, Walton HA. Epidemiological time series studies of PM2.5 and daily mortality and hospital admissions: a systematic review and meta-analysis. *Thorax* 2014; **69:** 660-5.

4 Atkinson RW, Mills IC, Walton HA, Anderson HR. Fine particle components and health--a systematic review and meta-analysis of epidemiological time series studies of daily mortality and hospital admissions. *J Expo Sci Environ Epidemiol* 2015; **25:** 208-14.

5 Atkinson RW, Butland BK, Anderson HR, Maynard RL. Long-term Concentrations of Nitrogen Dioxide and Mortality: A Meta-analysis of Cohort Studies. *Epidemiology* 2018; **29:** 460-72.

6 Burgan O, Smargiassi A, Perron S, Kosatsky T. Cardiovascular effects of sub-daily levels of ambient fine particles: a systematic review. *Environ Health* 2010; **9:** 26.

7 Cai Y, Zhang B, Ke W*, et al.* Associations of Short-Term and Long-Term Exposure to Ambient Air Pollutants With Hypertension: A Systematic Review and Meta-Analysis. *Hypertension* 2016; **68:** 62-70.

8 Cai X, Li Z, Scott EM, Li X, Tang M. Short-term effects of atmospheric particulate matter on myocardial infarction: a cumulative meta-analysis. *Environ Sci Pollut Res Int* 2016; **23:** 6139-48.

9 Chen J, Hoek G. Long-term exposure to PM and all-cause and cause-specific mortality: A systematic review and meta-analysis. *Environ Int* 2020; **143:** 105974.

10 Fajersztajn L, Saldiva P, Pereira LAA, Leite VF, Buehler AM. Short-term effects of fine particulate matter pollution on daily health events in Latin America: a systematic review and meta-analysis. *Int J Public Health* 2017; **62:** 729-38.

11 Farhadi Z, Abulghasem Gorgi H, Shabaninejad H, Aghajani Delavar M, Torani S. Association between PM. *BMC Public Health* 2020; **20:** 314.

12 Faustini A, Rapp R, Forastiere F. Nitrogen dioxide and mortality: review and meta-analysis of long-term studies. *Eur Respir J* 2014; **44:** 744-53.

13 Fu P, Guo X, Cheung FMH, Yung KKL. The association between PM. *Sci Total Environ* 2019; **655:** 1240-8.

14 Hoek G, Krishnan RM, Beelen R, Peters A, Ostro B, Brunekreef B, Kaufman JD. Long-term air pollution exposure and cardio- respiratory mortality: a review. *Environ Health* 2013; **12:** 43.

15 Jaganathan S, Jaacks LM, Magsumbol M*, et al.* Association of Long-Term Exposure to Fine Particulate Matter and Cardio-Metabolic Diseases in Low- and Middle-Income Countries: A Systematic Review. *Int J Environ Res Public Health* 2019; **16**.

16 Jilani MH, Simon-Friedt B, Yahya T*, et al.* Associations between particulate matter air pollution, presence and progression of subclinical coronary and carotid atherosclerosis: A systematic review. *Atherosclerosis* 2020; **306:** 22-32.

17 Kirrane EF, Luben TJ, Benson A*, et al.* A systematic review of cardiovascular responses associated with ambient black carbon and fine particulate matter. *Environment international* 2019; **127:** 305-16.

18 Lai HK, Tsang H, Wong CM. Meta-analysis of adverse health effects due to air pollution in Chinese populations. *BMC Public Health* 2013; **13:** 360.

19 Li XY, Yu XB, Liang WW*, et al.* Meta-analysis of association between particulate matter and stroke attack. *CNS Neurosci Ther* 2012; **18:** 501-8.

20 Liang R, Zhang B, Zhao X, Ruan Y, Lian H, Fan Z. Effect of exposure to PM2.5 on blood pressure: a systematic review and meta-analysis. *J Hypertens* 2014; **32:** 2130-40; discussion 41.

21 Liu X, Lian H, Ruan Y, Liang R, Zhao X, Routledge M, Fan Z. Association of Exposure to particular matter and Carotid Intima-Media Thickness: A Systematic Review and Meta-Analysis. *Int J Environ Res Public Health* 2015; **12:** 12924-40.

22 Liu Z, Wang F, Li W*, et al.* Does utilizing WHO's interim targets further reduce the risk - meta-analysis on ambient particulate matter pollution and mortality of cardiovascular diseases? *Environ Pollut* 2018; **242:** 1299-307.

23 Luben TJ, Nichols JL, Dutton SJ*, et al.* A systematic review of cardiovascular emergency department visits, hospital admissions and mortality associated with ambient black carbon. *Environ Int* 2017; **107:** 154-62.

24 Luo C, Zhu X, Yao C, Hou L, Zhang J, Cao J, Wang A. Short-term exposure to particulate air pollution and risk of myocardial infarction: a systematic review and meta-analysis. *Environ Sci Pollut Res Int* 2015; **22:** 14651-62.

25 Ma Y, Sun M, Liang Q*, et al.* The relationship between long-term exposure to PM. *Ecotoxicol Environ Saf* 2021; **208:** 111492.

26 Magalhaes S, Baumgartner J, Weichenthal S. Impacts of exposure to black carbon, elemental carbon, and ultrafine particles from indoor and outdoor sources on blood pressure in adults: A review of epidemiological evidence. *Environ Res* 2018; **161:** 345-53.

27 Mills IC, Atkinson RW, Kang S, Walton H, Anderson HR. Quantitative systematic review of the associations between short-term exposure to nitrogen dioxide and mortality and hospital admissions. *BMJ Open* 2015; **5:** e006946.

28 Mills IC, Atkinson RW, Anderson HR, Maynard RL, Strachan DP. Distinguishing the associations between daily mortality and hospital admissions and nitrogen dioxide from those of particulate matter: a systematic review and meta-analysis. *BMJ Open* 2016; **6:** e010751.

29 Mustafic H, Jabre P, Caussin C*, et al.* Main air pollutants and myocardial infarction: a systematic review and meta-analysis. *Jama* 2012; **307:** 713-21.

30 Newell K, Kartsonaki C, Lam KBH, Kurmi O. Cardiorespiratory health effects of gaseous ambient air pollution exposure in low and middle income countries: a systematic review and meta-analysis. *Environ Health* 2018; **17:** 41.

31 Niu Z, Liu F, Yu H, Wu S, Xiang H. Association between exposure to ambient air pollution and hospital admission, incidence, and mortality of stroke: an updated systematic review and meta-analysis of more than 23 million participants. *Environ Health Prev Med* 2021; **26:** 15.

32 Pranata R, Vania R, Tondas AE, Setianto B, Santoso A. A time-to-event analysis on air pollutants with the risk of cardiovascular disease and mortality: A systematic review and meta-analysis of 84 cohort studies. *J Evid Based Med* 2020; **13:** 102-15.

33 Provost EB, Madhloum N, Int Panis L, De Boever P, Nawrot TS. Carotid intima-media thickness, a marker of subclinical atherosclerosis, and particulate air pollution exposure: the meta-analytical evidence. *PLoS One* 2015; **10:** e0127014.

34 Sangkharat K, Fisher P, Thomas GN, Thornes J, Pope FD. The impact of air pollutants on ambulance dispatches: A systematic review and meta-analysis of acute effects. *Environ Pollut* 2019; **254:** 112769.

35 Scheers H, Jacobs L, Casas L, Nemery B, Nawrot TS. Long-Term Exposure to Particulate Matter Air Pollution Is a Risk Factor for Stroke: Meta-Analytical Evidence. *Stroke* 2015; **46:** 3058-66.

36 Shah AS, Langrish JP, Nair H*, et al.* Global association of air pollution and heart failure: a systematic review and meta-analysis. *Lancet* 2013; **382:** 1039-48.

37 Shah AS, Lee KK, McAllister DA*, et al.* Short term exposure to air pollution and stroke: systematic review and meta-analysis. *BMJ* 2015; **350:** h1295.

38 Shao Q, Liu T, Korantzopoulos P, Zhang Z, Zhao J, Li G. Association between air pollution and development of atrial fibrillation: A meta-analysis of observational studies. *Heart Lung* 2016; **45:** 557-62.

39 Shin HH, Fann N, Burnett RT, Cohen A, Hubbell BJ. Outdoor fine particles and nonfatal strokes: systematic review and meta-analysis. *Epidemiology* 2014; **25:** 835-42.

40 Song X, Liu Y, Hu Y, Zhao X, Tian J, Ding G, Wang S. Short-Term Exposure to Air Pollution and Cardiac Arrhythmia: A Meta-Analysis and Systematic Review. *Int J Environ Res Public Health* 2016; **13**.

41 Stieb DM, Zheng C, Salama D*, et al.* Systematic review and meta-analysis of case-crossover and time-series studies of short term outdoor nitrogen dioxide exposure and ischemic heart disease morbidity. *Environ Health* 2020; **19:** 47.

42 Teng TH, Williams TA, Bremner A*, et al.* A systematic review of air pollution and incidence of out-of-hospital cardiac arrest. *J Epidemiol Community Health* 2014; **68:** 37-43.

43 Wang Y, Eliot MN, Wellenius GA. Short-term changes in ambient particulate matter and risk of stroke: a systematic review and meta-analysis. *J Am Heart Assoc* 2014; **3**.

44 Yamamoto SS, Phalkey R, Malik AA. A systematic review of air pollution as a risk factor for cardiovascular disease in South Asia: limited evidence from India and Pakistan. *Int J Hyg Environ Health* 2014; **217:** 133-44.

45 Yang WS, Wang X, Deng Q, Fan WY, Wang WY. An evidence-based appraisal of global association between air pollution and risk of stroke. *Int J Cardiol* 2014; **175:** 307-13.

46 Yang BY, Qian Z, Howard SW, Vaughn MG, Fan SJ, Liu KK, Dong GH. Global association between ambient air pollution and blood pressure: A systematic review and meta-analysis. *Environ Pollut* 2018; **235:** 576-88.

47 Yang H, Li S, Sun L*, et al.* Smog and risk of overall and type-specific cardiovascular diseases: A pooled analysis of 53 cohort studies with 21.09 million participants. *Environ Res* 2019; **172:** 375-83.

48 Yu XB, Su JW, Li XY, Chen G. Short-term effects of particulate matter on stroke attack: meta-regression and meta-analyses. *PLoS One* 2014; **9:** e95682.

49 Yuan S, Wang J, Jiang Q*, et al.* Long-term exposure to PM(2.5) and stroke: A systematic review and meta-analysis of cohort studies. *Environ Res* 2019; **177:** 108587.

50 Yue C, Yang F, Wang L, Li F, Chen Y. Association between fine particulate matter and atrial fibrillation in implantable cardioverter defibrillator patients: a systematic review and meta-analysis. *J Interv Card Electrophysiol* 2020; **59:** 595-601.

51 Yue C, Yang F, Li F, Chen Y. Association between air pollutants and atrial fibrillation in general population: A systematic review and meta-analysis. *Ecotoxicol Environ Saf* 2021; **208:** 111508.

52 Zanoli L, Lentini P, Granata A*, et al.* A systematic review of arterial stiffness, wave reflection and air pollution. *Mol Med Rep* 2017; **15:** 3425-9.

53 Zhao R, Chen S, Wang W, Huang J, Wang K, Liu L, Wei S. The impact of short-term exposure to air pollutants on the onset of out-of-hospital cardiac arrest: A systematic review and meta-analysis. *Int J Cardiol* 2017; **226:** 110-7.

54 Zhang S, Routledge MN. The contribution of PM(2.5) to cardiovascular disease in China. *Environ Sci Pollut Res Int* 2020; **27:** 37502-13.

55 Zhao L, Liang HR, Chen FY, Chen Z, Guan WJ, Li JH. Association between air pollution and cardiovascular mortality in China: a systematic review and meta-analysis. *Oncotarget* 2017; **8:** 66438-48.

56 Zhu W, Cai J, Hu Y, Zhang H, Han X, Zheng H, Wu J. Long-term exposure to fine particulate matter relates with incident myocardial infarction (MI) risks and post-MI mortality: A meta-analysis. *Chemosphere* 2021; **267:** 128903.
